# MARCKS mediates vascular contractility through regulating interactions between voltage-gated Ca^2+^ channels and PIP_2_

**DOI:** 10.1016/j.vph.2020.106776

**Published:** 2020-09

**Authors:** Kazi S. Jahan, Jian Shi, Harry Z.E. Greenberg, Sam Khavandi, Miguel Martín-Aragón Baudel, Vincenzo Barrese, Iain A. Greenwood, Anthony P. Albert

**Affiliations:** a Vascular Biology Research Centre, Molecular and Clinical Research Institute, St. George's, University of London, Cranmer Terrace, London SW17 0RE, UK; b Leeds Institute of Cardiovascular and Metabolic Medicine, Faculty of Medicine and Health, University of Leeds, Leeds LS2 9JT, UK; c Department of Pharmacology, University of California, 451, Health Sciences Drive, Suite 3503, Davis, CA 95615, USA; d Department of Neurosciences, Reproductive Sciences and Dentistry, University of Naples Federico II, Corso Umberto I, 40, 80138 Napoli, NA, Italy

**Keywords:** MARCKS, PIP_2_, Voltage-gated Ca^2+^ channels, Contractility

## Abstract

Phosphatidylinositol 4,5-bisphosphate (PIP_2_) acts as substrate and unmodified ligand for Gq-protein-coupled receptor signalling in vascular smooth muscle cells (VSMCs) that is central for initiating contractility. The present work investigated how PIP_2_ might perform these two potentially conflicting roles by studying the effect of myristoylated alanine-rich C kinase substrate (MARCKS), a PIP_2_-binding protein, on vascular contractility in rat and mouse mesenteric arteries. Using wire myography, MANS peptide (MANS), a MARCKS inhibitor, produced robust contractions with a pharmacological profile suggesting a predominantly role for L-type (CaV1.2) voltage-gated Ca^2+^ channels (VGCC). Knockdown of MARCKS using morpholino oligonucleotides reduced contractions induced by MANS and stimulation of α_1_-adrenoceptors and thromboxane receptors with methoxamine (MO) and U46619 respectively. Immunocytochemistry and proximity ligation assays demonstrated that MARCKS and CaV1.2 proteins co-localise at the plasma membrane in unstimulated tissue, and that MANS and MO reduced these interactions and induced translocation of MARCKS from the plasma membrane to the cytosol. Dot-blots revealed greater PIP_2_ binding to MARCKS than CaV1.2 in unstimulated tissue, with this binding profile reversed following stimulation by MANS and MO. MANS evoked an increase in peak amplitude and shifted the activation curve to more negative membrane potentials of whole-cell voltage-gated Ca^2+^ currents, which were prevented by depleting PIP_2_ levels with wortmannin. This present study indicates for the first time that MARCKS is important regulating vascular contractility and suggests that disinhibition of MARCKS by MANS or vasoconstrictors may induce contraction through releasing PIP_2_ into the local environment where it increases voltage-gated Ca^2+^ channel activity.

## Graphical abstract

**Figure:**
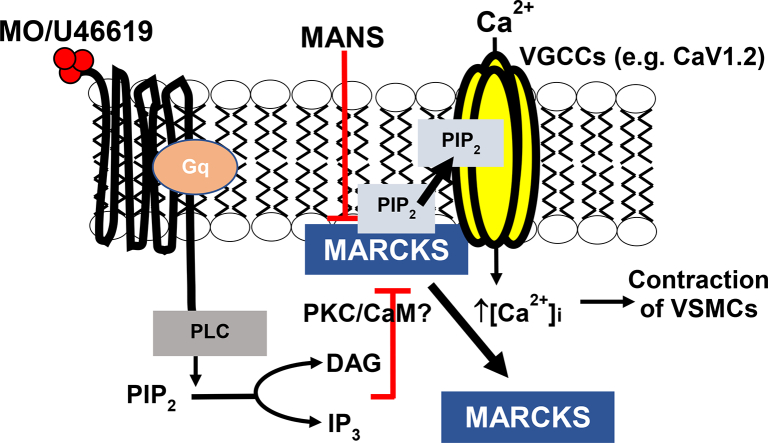

## 1 Introduction

It is well-established that phosphatidylinositol 4,5-bisphosphate (PIP_2_) acting as a substrate for Gq-protein-coupled receptor signalling in vascular smooth muscle cells (VSMCs) has a central role in vasoconstrictor-mediated contractility [1,2]. Gq-protein receptor-mediated phospholipase C (PLC) activity leads to PIP_2_ hydrolysis and generation of inositol 1,4,5-trisphosphate (IP_3_) and diacylglycerol (DAG) which drive multiple pathways that increase intracellular Ca^2+^ concentration to induce contraction. In particular, IP_3_-mediated Ca^2+^ release from sarcoplasmic reticulum stores and DAG-mediated signal transduction pathways regulate an array of cation, Cl^−^, and K^+^ channel subtypes to induce membrane depolarisation and activation of voltage-gated Ca^2+^ channels (VGCC) to produce Ca^2+^ influx and contraction [1,2].

There is also considerable evidence that, in addition to its classical role as a substrate for Gq-mediated PLC activity, PIP_2_ acts as an unmodified ligand to regulate proteins involved in modulating vascular contractility including ion channels involved in regulating membrane potential and VGCC activity [[3], [4], [5], [6], [7], [8], [9], [10]]. This raises an important question in vascular biology; how can PIP_2_ act as both substrate and unmodified ligand to regulate different cellular pathways involved in regulating contractility? An explanation is the existence of independent pools of PIP_2_, produced through localised formation and/or sequestration of PIP_2_ at the plasma membrane [11]. Sequestration is an attractive hypothesis as this would allow PIP_2_ to be retained in the local environment, thus preventing locally formed PIP_2_ from rapidly diffusing away from its site of action [11,12]. There are several natively unfolded proteins which permit electrostatic interactions with PIP_2_ and therefore sequestration, such as myristoylated alanine-rich C kinase (MARCKS), growth-associated protein 43 (GAP43), and cytoskeleton-associated protein 23 (CAP23) [13,14]. These proteins are proposed to act as PIP_2_ buffers or PIPmodulins to release PIP_2_ into the local environment following stimulation, allowing this source of PIP_2_ to act as an unmodified ligand [11]. Hence PIP_2_ sequestration proteins may have important roles in regulating vascular contractility by controlling PIP_2_-mediated cellular processes. To date there have been no studies on the effect of PIP_2_ sequestration proteins on vascular contraction, and therefore the present study investigates the role of MARCKS in such a function. MARCKS was chosen for this study since it is a ubiquitously expressed protein whereas GAP43 and CAP23 are mainly found in neurones [13,14].

Much is known about the chemical properties and cellular processes that regulate MARCKS but relatively little is known about the function of this PIP_2_-binding protein, although it has been associated with neuronal development, cell migration and proliferation, and secretary pathways [[15], [16], [17], [18], [19], [20], [21], [22], [23], [24], [25]]. MARCKS structure contains two important regions, a myristoylated N-terminal region which weakly anchors it to the plasma membrane, and an effector domain containing a sequence of basic amino acids which form electrostatic interactions with PIP_2_ that provide further stability at the plasma membrane. The effector domain also acts as a protein kinase (PKC) substrate and a calmodulin (CaM)-binding region, with PKC-dependent phosphorylation and CaM binding both reducing electrostatic interactions with PIP_2_, leading to PIP_2_ release into the local environment and MARCKS to be translocated to the cytosol. These properties define MARCKS as a reversible PIP_2_ buffer, which can provide spatial sequestration and release of PIP_2_ to allow targeted function.

Several studies have shown that MARCKS is expressed in VSMCs where it has been proposed to have diverse functions including regulating PKC and CaM signalling [26], upregulation in neointima hyperplasia involving cell migration and proliferation [[27], [28], [29]], and modulation of TRPC1 channel activity [30]. However, there have been no studies on the role of MARCKS in regulating vascular contractility, and therefore this was the aim of the present work. To achieve this, we investigated the effect of the selective MARCKS inhibitor, MANS peptide (MANS), and knockdown of MARCKS expression using morpholino oligonucleotide technology [31,32]. MANS is a 24 amino acid sequence that corresponds to the initial N-terminal myristoylated region of MARCKS [33,34]. As such the MANS competes with endogenous MARCKS for binding to the plasma membrane, which leads to MARCKS being translocated into the cytosol and whilst releasing PIP_2_ into the local environment. In addition, the hydrophobic myristate moiety means MANS is highly cell permeant. MANS has been used in several studies to reveal the role of MARCKS in mediating mucus secretion in the airways [33,34], immune cell degranulation [35,36], amylase release [37], and lung cancer metastasis [38].

The present study provides the first evidence that MARCKS acting as a plasma membrane PIP_2_ buffer has an important role in regulating vascular contractility. Our findings suggest that disinhibition of MARCKS by MANS or vasoconstrictors may induce contraction through releasing PIP_2_ into the local environment where it increases voltage-gated Ca^2+^ channel activity. These hypotheses provide provocative novel ideas on cellular mechanisms governing vascular contraction, which are likely to have important implications for understanding physiological and pathological processes.

## 2 Methods

An expanded Methods sections is provided in the supplementary data.

### 2.1 Animals

All animal procedures were carried out in accordance with guidelines laid down by St George's, University of London Animal Welfare Committee and conform with the principles and regulations described by the Service Project Licence: 70/8512. Male Wistar rats (8–12 weeks) and 129-SV mice (6–9 weeks) were used for the purpose of this study. Rats were supplied from Charles River, UK and 129-SV mice were bred in the Biological Research Facility at St George's, University of London. Animals were housed and maintained in standard sized plastic cages, with a 12 h light-dark cycle, ambient room temperature of 18–20 °C, relative humidity of approximately 50%, and water and lab rodent diet (Specialist Dietary Services, UK) available ad libitum. Animals were culled by cervical dislocation in accordance with the UK Animals Scientific Procedures Act of 1986 and as revised by European Directive 2010/63/EU. Mesenteric arteries were dissected and cleaned of adherent fat in physiological salt solution containing (mM): 126 NaCl, 6 KCl, 10 Glucose, 11 HEPES, 1.2 MgCl_2_, and 1.5 CaCl_2_, with pH adjusted to 7.2 with 10 M NaOH. Mouse mesenteric arteries were used for wire myography, mouse IP_3_ ELISA assay and proximity ligation assays. Rat mesenteric arteries were used when a greater yield of protein from tissue lysates or single VSMCs following tissue dispersal were required for better experimental efficiency such as transfection for imaging PLC activity, dot-blots and electrophysiological recordings.

### 2.2 Western blotting

Mouse and rat mesenteric arteries were homogenised with radio immunoprecipitation assay lysis buffer containing a protease inhibitor cocktail (Santa Cruz, USA) (see supplementary data for more details). Samples were then loaded onto SDS-PAGE gels (4–12% Bis-Tris, Invitrogen, UK), subjected to electrophoresis, and then transferred onto a polyvinylidene fluoride membrane (Amersham Biosciences, UK). The membrane was then probed with an anti-MARCKS antibody (1:100; SC-6455, Santa Cruz, USA). Protein bands were visualized with a horseradish peroxidase-conjugated secondary antibody and enhanced chemiluminescence reagents (Pierce Biotechnology, USA) for 1 min and exposed to photographic films (Amersham Biosciences, UK).

### 2.3 Immunocytochemistry

Freshly dispersed VSMCs (see supplementary data for more details) were fixed with 4% (*w*/*v*) paraformaldehyde for 15 min and permeabilised with PBS containing 0.25% (*v*/v) Triton X-100 for 10 min at room temperature. Cells were then treated with phosphate-buffered saline (PBS) containing 1% (*w*/*v*) bovine serum albumin (BSA) for 1 h at room temperature, to block non-specific binding of antibodies. Immunostaining was performed using an anti-MARCKS primary antibody (1:100; SC-6455, Santa Cruz, USA) and/or anti-CaV1.2 primary antibody (1:100; ACC-003, Alomone, Israel) overnight at 4 °C. Cells were then washed and incubated with a 488 fluorophore-conjugated donkey anti-goat secondary antibody (1:1000; A-11055, Alexa Fluor, UK) for 1 h at room temperature. Unbound secondary antibodies were removed by washing with PBS, and nuclei were labelled with 4, 6-diamidino-2-phenylindole (DAPI) mounting medium (Sigma, UK). Control experiments were performed by replacing primary antibody with goat serum (1:100; Sigma, UK) or omitting either primary or secondary antibodies. Cells were imaged using a Zeiss LSM 510 laser scanning confocal microscope (Carl Zeiss, Germany).

### 2.4 Isometric tension recordings

Segments of mouse superior mesenteric artery of about 2 mm in length were mounted on a wire myograph (Danish Myo Technology, Denmark) and endothelium was removed by rubbing the intima with a human hair. Vessel segments were bathed in Krebs solution containing (mM): 118.4 NaCl, 4.69 KCl, 1.18 MgSO_4_, 1.22 KH_2_PO_4_, 25 NaHCO_3_, 10 glucose and 2 CaCl_2_, maintained at 37 °C and constantly aerated with 95% O_2_ and 5% CO_2_. Vessel segments were then normalised to 90% of the internal circumference predicted to occur under a transmural pressure of 100 mmHg [39]. Vessel segments were then equilibrated for 30 min and assessed for vessel viability with 60 mM KCl for 5 min. Endothelium integrity was then assessed by stably pre-contracting vessels with 10 μM methoxamine (MO) followed by 10 μM carbachol (CCh). Carbachol-induced relaxation of ˂10% indicated successful removal of the endothelium. Vessels were then equilibrated for 10 min before the experimental protocol (see supplementary data for more details).

### 2.5 Morpholino-mediated MARCKS knockdown

10 μM MARCKS-targeted (5’-GCACCCATGCTGGCTTCTTCAACAA-3′) or scrambled morpholino (5’-GCACCgATcCTcGCTTgTTgAACAA) oligonucleotides (Gene Tools Inc., USA) were mixed with Lipofectamine 2000 (Life Technologies, UK) in Opti-MEM (Life Technologies, UK) and left at room temperature for 2 h. The Opti-MEM mix was then added to Dulbecco's modified Eagle's medium (DMEM)/Nutrient Mixture F-12 (Life Technologies, UK), containing 1% Penicillin-Streptomycin (Sigma, UK), and mouse superior mesenteric arteries were placed in this solution at 37 °C for 48 h. Successful delivery of morpholino antisense oligonucleotides was assessed using an Olympus 1 × 60 fluorescence inverted microscope (Olympus, UK) with a Hamamatsu C4742–95 digital camera and motorized stage (Hamamatsu Protonics, UK). Successful knock-down of the protein and selectivity of MARCKS-targeted morpholino oligonucleotides were assessed by western blotting and immunocytochemical staining.

### 2.6 Transfection of PIP_2_ biosensors

GFP-PLCδ-PH was transfected into freshly dispersed rat mesenteric artery VSMCs by electroporation using Nucleofector™ Technology (Lonza, USA) as per manufacturer's instructions (see supplementary data for more details). Following electroporation, cells were incubated at 37 °C in 95% O_2_ and 5% CO_2_ in a humidified incubator for 48 h before being imaged. Transfected cells were imaged at 37 °C in 95% O_2_ and 5% CO_2_ in a humidified chamber using a Nikon AR1 inverted confocal microscope and associated software (Nikon Instruments, UK). Excitation was produced by a 488 laser. Final images were produced using PowerPoint (Microsoft XP; Microsoft, USA). Cell culture media contained: Ca^2+^ free DMEM supplemented with 1% fetal bovine serum (FBS), 1% Penicillin-Streptomycin, 2.5 mM l-Glutamine, 1 mM sodium pyruvate and 1 μM wortmannin. 1% FBS was used to maintain VSMC contractile phenotype and 1 μM wortmannin was used to prevent contraction of VSMCs following pre-treatment with MANS or MO, which prevents accurate imaging (as shown previously) [40,41].

### 2.7 Dot-blots

Rat mesenteric artery segments were dissected, divided into three, and pre-treated with distilled water, 100 μM MANS, or 10 μM MO for 20 min at room temperature before extraction of protein (see supplementary data for more details). Next, 500 μg of tissue lysate was immunoprecipitated (see supplementary data for more details) with either an anti-MARCKS (SC-6455, Santa Cruz, USA) or anti-CaV1.2 primary antibody (ACC-003, Alomone, Israel). Then, 15 μl of immunoprecipitated rat mesenteric artery tissue lysate was blotted on nitrocellulose membranes (Amersham Biosciences, UK) and allowed to dry before being blocked in 5% (*w*/*v*) milk powder in 0.05% (*v*/v) PBST. Membranes were then incubated with an anti-PIP_2_ antibody (1:200; SC-53412, Santa Cruz, USA) overnight at 4 °C. Visualisation was performed with a donkey anti-mouse (1:10,000; LI-COR Biotechnology, UK) fluorescently-conjugated secondary antibody, and imaged on the Odyssey Infrared Imaging System (LI-COR Biotechnology, UK). Blot intensities were analyzed with Image Studio, (version 3.0; LI-COR Biotechnology, UK).

### 2.8 Whole-cell recording

Whole-cell patch clamp voltage-clamp and current clamp recordings were conducted on freshly dispersed rat mesenteric artery VSMCs. In voltage-clamp studies, VGCC activity was evoked by applying 300 ms voltage steps from −80 mV to +40 mV at 10 mV intervals every 30 s from a holding potential of −60 mV. A control current-voltage (I/V) relationship curve was recorded before 100 μM MANS peptide was added to the extracellular solution in the presence or absence of 20 μM wortmannin, and then 3 μM nicardipine was used to confirm VGCC channel activity. The extracellular solution contained (mM): 110 NaCl, 1 CsCl, 10 BaCl_2_, 1.2 MgCl_2_, 10 glucose, 10 HEPES, 0.1 DIDS, 0.1 GdCl_2_, adjusted to pH 7.4 with 10 M NaOH. The internal patch pipette solution contained (mM): 135 CsCl, 2.5 Mg-ATP, 0.1 GTP, 10 HEPES, 10 EGTA, adjusted to pH 7.2 with 10 M CsOH. In current clamp studies, membrane potential was recorded using an extracellular solution contained (mM): 126 NaCl, 6 KCl, 10 Glucose, 11 HEPES, 1.2 MgCl_2_, and 1.5 CaCl_2_, with pH adjusted to 7.2 with 10 M NaOH and an internal patch pipette solution contained (mM): 126 KCl, 5 NaCl, 2.5 Mg-ATP, 0.1 GTP, 10 HEPES, 1 BAPTA, adjusted to pH 7.2 with 10 M CsOH. Both voltage-clamp and current clamp recordings were conducted once the access resistance was <20 MΩ, filtered at 1 kHz, and sampled at 5 kHz. All recordings were made at room temperature.

### 2.9 Data analysis

All data is expressed as mean ± standard error of mean for corresponding number (*n*) of animals. All statistical analysis was conducted using GraphPad Prism software (Version 7.04, GraphPad, USA). A *P* value of less than 5% (*P* < .05) was considered statistically significant.

### 2.10 Materials

All chemicals and drugs were purchased from Sigma-Aldrich (Sigma Chemical Co., Poole, UK) or Tocris (Tocris Biosciences, Bristol, UK). MANS peptide (MANS) (Genemed Synthesis, USA) is a cell-permeable synthetic peptide that is identical to the first 24 amino acids of the MARCKS N-terminus [33,34] and contains the N-terminus myristic moiety (MA-GAQFSKTAAKGEAAAERPGEAAVA, MA = N-terminal myristate). GFP-PLCδ-PH was a gift from Professor Tobias Meyer (Plasmid identification #21179; Addgene, USA). Drugs were dissolved in distilled water or dimethyl sulfoxide (DMSO).

## 3 Results

### 3.1 MARCKS is expressed in mesenteric artery VSMCs

**Fig. 1:**
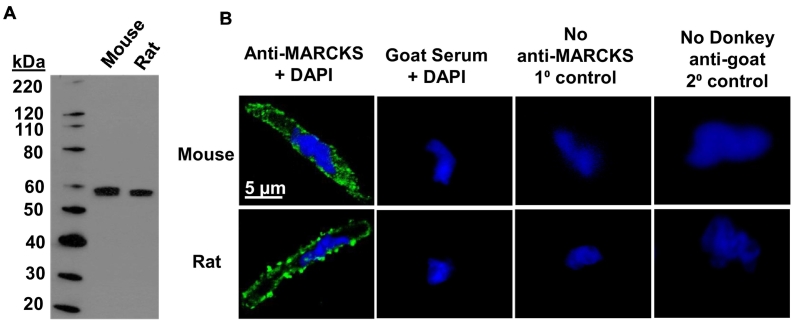
Expression of MARCKS in mouse and rat mesenteric artery. A, Representative western blot for MARCKS expression from tissue lysates of mouse and rat mesenteric arteries using an anti-MARCKS antibody. B, Immunostaining of single freshly isolated mouse and rat mesenteric artery VSMCs labelled with the same anti-MARCKS antibody as used in A. Control experiments were performed by replacing anti-MARCKS antibody with goat serum, or by omitting anti-MARCKS or donkey anti-goat antibodies. Immunoblots are representative of *N* *=* 3 experimental preparations using *n* = 3 animals per preparation, and immunocytochemical images are representative of data from n *=* 3 animals with *N* ≥ 3 cells per animal.

In our initial experiments we investigated the expression of MARCKS in tissue lysates and freshly isolated VSMCs from mouse and rat mesenteric arteries. Fig. 1A shows that western blot analysis revealed a single protein band of about 60 kDa following immunoblotting with an anti-MARCKS antibody, and Fig. 1B illustrates that distribution of MARCKS staining using the same anti-MARCKS antibody was predominantly located at, or close to, the plasma membrane of VSMCs using immunocytochemstry. These findings indicate that MARCKS is expressed in mesenteric artery, and that it may have a functional role at the plasma membrane of VSMCs.

### 3.2 MANS peptide induces vascular contractility

**Fig. 2:**
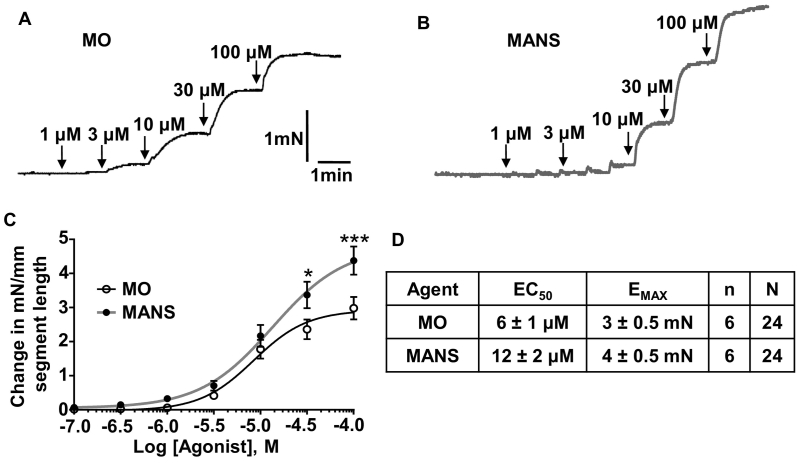
Effects of methoxamine (MO) and MANS on contractility of mouse mesenteric artery. A and B, Representative traces and C, mean concentration-effect curves of MO or MANS on artery segments. D, Table comparing mean EC_50_ and E_MAX_ values of MO- and MANS-induced contractions. Data from *n* = 6 animals, with *N* = 4 vessel segments per animal. Two-way ANOVA followed by Bonferroni Post-hoc. ^⁎^*P* < .05; ^⁎⁎⁎^*P* < .001.

To investigate the role of MARCKS on vascular contractility we compared the effect of the selective MARCKS inhibitor, MANS peptide (MANS, see Introduction and Methods for peptide details) [33,34] with the α_1_-adrenoceptor agonist methoxoamine (MO) on isometric tension recordings from segments of mouse mesenteric artery using wire myography. Fig. 2 shows that bath applications of MO and MANS induced concentration-dependent increases in contractility, with MANS having a greater effective half maximal concentration (EC_50_) and maximal effect (E_Max_) than MO of about 2-fold and 30% respectively. Contractile responses to both MO and MANS were sustained during continued application for 30 min and were reproducible following multiple cycles of bath application and washing (Fig. S1). These results suggest that MARCKS exerts an inhibitory action on contraction in unstimulated vessels, and that removal of this inhibition action by MANS induces vascular contractility in the absence of any receptor stimulation. The potential physiological importance of MARCKS on contractility is highlighted by the equivalence of contractions produced by MANS and stimulation of the α_1_-adrenoceptor-mediated vasoconstrictor pathway by MO.

### 3.3 Effect of reducing MARCKS expression on MANS- and vasoconstrictor-evoked contractility

To investigate the selectivity of MANS and provide further evidence that MARCKS regulates vascular contractility, we examined the effect of reducing MARCKS expression on MANS- and vasoconstrictor-evoked contractility using morpholino oligonucleotide technology previously used to investigate other proteins in vascular contractility [31,32] (see Methods for oligomer details).

In initial experiments, we tested whether MARCKS-targeted morpholino oligomers reduce MARCKS expression. Fig. S2 shows that fluorescein-tagged morpholino oligonucleotides were successfully transfected into segments of mouse mesenteric artery after 48 h, and that tissue lysates from vessels pre-treated with MARCKS-targeted compared to scrambled sequence oligomers had significantly reduced MARCKS expression by over 50%. Fig. S4 also shows that distribution of MARCKS staining at, or close to, the plasma membrane of VSMCs was reduced following pre-treatment of vessels with MARCKS-targeted oligomers. In control experiments, Fig. S2 shows that expression of α-tubulin or total protein levels were not altered by MARCKS-targeted oligomers. In additional control experiments, we examined the effect of MARCKS-targeted oligomers on expression levels of L-type (CaV1.2) VGCCs, as activation of these channels are known to be important for initiating vascular contractility [[42], [43], [44], [45]]. Figs. S3 and S4 show that expression of CaV1.2 protein levels and distribution of CaV1.2 staining at, or close to, the plasma membrane of VSMCs was not altered in vessels pre-treated with MARCKS-targeted compared to scrambled oligomers. These results indicate that MARCKS-targeted oligomers produced substantial reduction of MARCKS expression but did alter α-tubulin, total protein and CaV1.2 expression levels.

**Fig. 3:**
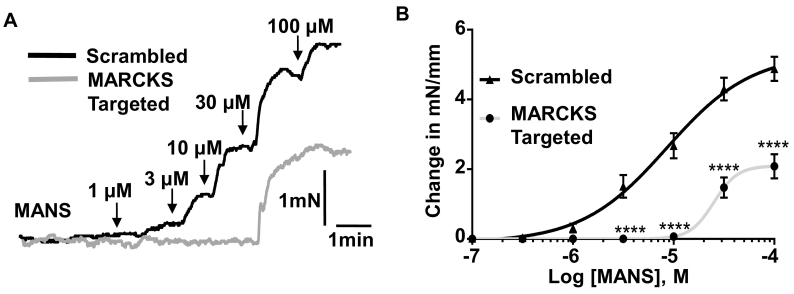
Effect of MARCKS knock-down on MANS-evoked contractions in mouse mesenteric arteries. A, Representative trace and B, mean concentration-effect curve showing attenuated effect of MANS-evoked contractions in artery segments transfected with MARCKS-targeted morpholino oligonucleotides compared with vessels pre-incubated with scrambled sequences. Data from *n* = 6 animals, with N ≥ 3 vessel segments per animal. Two-way ANOVA followed by Bonferroni Post-hoc. ^⁎⁎⁎⁎^*P* < .001.

Figs. 3 and S5 show that the MANS-evoked contractions of mouse mesenteric arteries pre-treated with scrambled oligomers for 48 h had similar mean EC_50_ and E_max_ values to those obtained from vessels recorded from on the same day of isolation (Fig. 2). Figs. 3 and S5 also show that, although the resting tension of mouse mesenteric artery segments was not altered with pre-treatment of MARCKS-targeted compared to scrambled oligomers, MANS-induced contractions were significantly reduced by MARCKS-targeted oligomers, with mean EC_50_ and E_max_ values increased by about 3-fold and reduced by over 50% respectively.

**Fig. 4:**
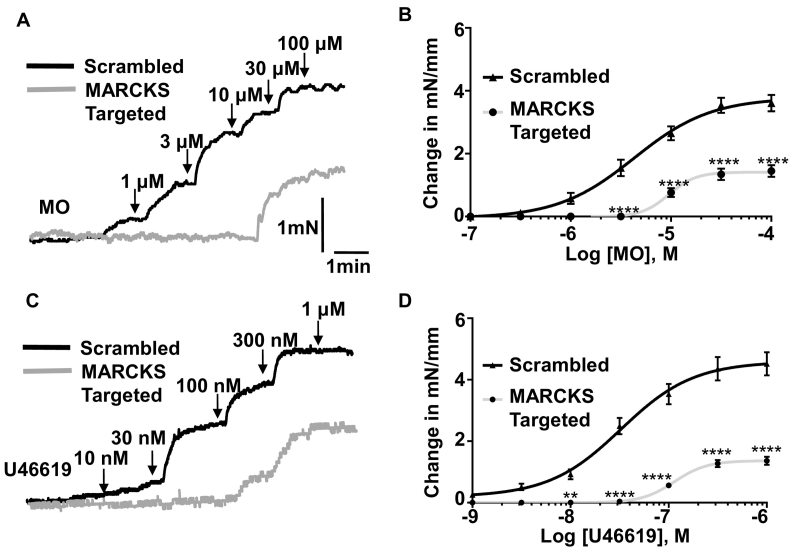
Effect of MARCKS knock-down on MANS- and methoxamine (MO)-evoked contractions in mouse mesenteric arteries. A and C, Representative traces and B and D, mean concentration-effect curves showing attenuated effect of MO- and U46619-evoked contractions in artery segments transfected with MARCKS-targeted morpholino oligonucleotides compared with vessels pre-incubated with scrambled sequences. Data from *n* = 6 animals, with N ≥ 3 vessel segments per animal. Two-way ANOVA followed by Bonferroni Post-hoc. ^⁎⁎⁎⁎^*P* < .001.

Interestingly, Figs. 4 and S5 also show that contractions of mouse mesenteric artery evoked by MO and the thromboxane receptor agonist U46619 were inhibited in vessels pre-treated with MARCKS-targeted compared to scrambled oligomers, with mean EC_50_ and E_max_ values increased by about 3-fold and reduced by over 50% respectively.

It is possible that MARCKS-targeted oligomers reduce contractility by inhibiting the activity of VGCCs and/or interfering with the Ca^2+^-dependent contractile apparatus involving Ca^2+^-CaM, myosin light chain kinase (MLCK), actin and myosin. Therefore, in control experiments, we investigated the effect of MARCK-targeted oligomers on contractions induced by high concentrations of KCl which induce contractility by producing membrane depolarisation, activation of VGCCs, Ca^2+^ influx and contraction and also by the Ca^2+^ ionophore ionomycin that causes Ca^2+^ influx independently of stimulation of plasmalemmal receptors or activation of VGCCs. Fig. S6 shows that contractions induced by bath application of 60 mM and 120 mM KCl and 3 μM ionomycin were similar in vessels pre-treated with MARCKS-targeted and scrambled oligomers. These findings indicate that knockdown of MARCKS is unlikely to reduce MANS- and vasoconstrictor-evoked contractility by blocking VGCC activity or decreasing the ability of vessels to contract.

These results provide compelling evidence that MANS increases vascular contractility by acting via MARCKS and indicates that MARCKS is likely to have an important role in vasoconstrictor-mediated contraction.

### 3.4 MANS-induced vascular contractility is inhibited by L- and T-type VGCC blockers

**Fig. 5:**
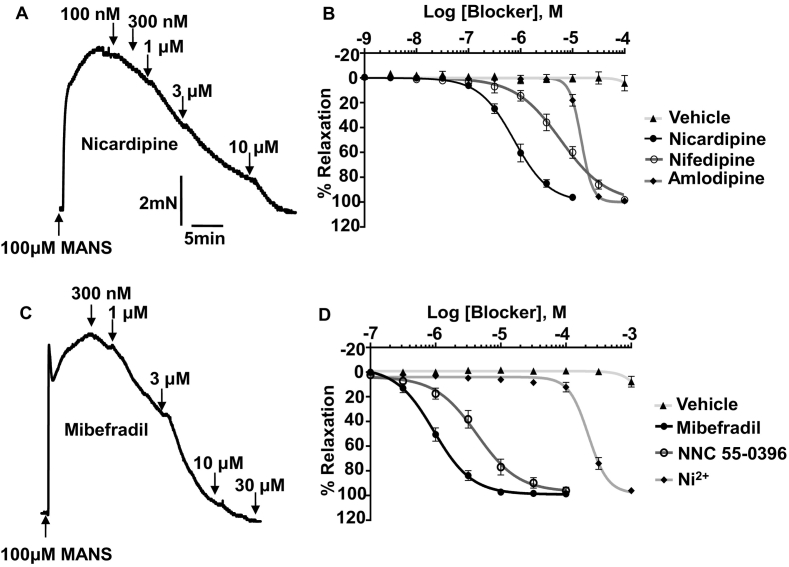
Effect of L-type and T-type VGCC blockers on mouse mesenteric arteries pre-contracted with MANS. A and C, Representative traces showing the effect of a L-type and T-type VGCC blockers on MANS pre-constricted tone respectively. B and D, Mean concentration-effect curves of L-type and T-type VGCC blockers on MANS precontracted tone respectively. Data from *n* = 6 animals, with N ≥ 3 vessel segments per animal.

In the next series of experiments, we investigated possible mechanisms involved in mediating MANS-induced contractions, to provide an insight into how MARCKS may regulate vascular contractility. We therefore investigated the effect of L- (CaV1.2) and T-type (CaV3.1/3.2 VGCC blockers on MANS-induced contraction of mouse mesenteric artery as both these VGCCs are thought to play a central role in mediating vascular contractility [[42], [43], [44], [45]]. Figs. 5 and S7 show that co-application of the L-type channel blockers nicardipine, nifedipine and amlodipine or the T-type channel blockers mibefradil, NNC 55–0396 and Ni^2+^ produced concentration-dependent inhibition of pre-contracted vascular tone induced by a near maximal concentration of MANS (100 μM). All blockers could produce 100% relaxation. These findings suggest that vascular contractility induced by inhibition of MARCKS requires activation of VGCCs, which may involve both L- and T-type channel subtypes.

### 3.5 MANS has little effect on PLC activity or membrane potential

A potential hypothesis to explain why MANS induces contractility via VGCCs is that by inhibiting MARCKS it causes MARCKS to release PIP_2_, which then is available to drive PLC activity and subsequent downstream stimulation of VGCCs. We explored this idea by studying the effect of MANS on PLC activity by transfecting rat mesenteric artery VSMCs primary cultured in low serum conditions (see Methods) with GFP-PLCδ-PH, a fluorescent biosensor with a high affinity for PIP_2_ and IP_3_ [46] and then recording signal changes in fluorescent intensity units at, or close to, the plasma membrane (Fm) and within the cytosol (Fc) as previously described [40,41].

**Fig. 6:**
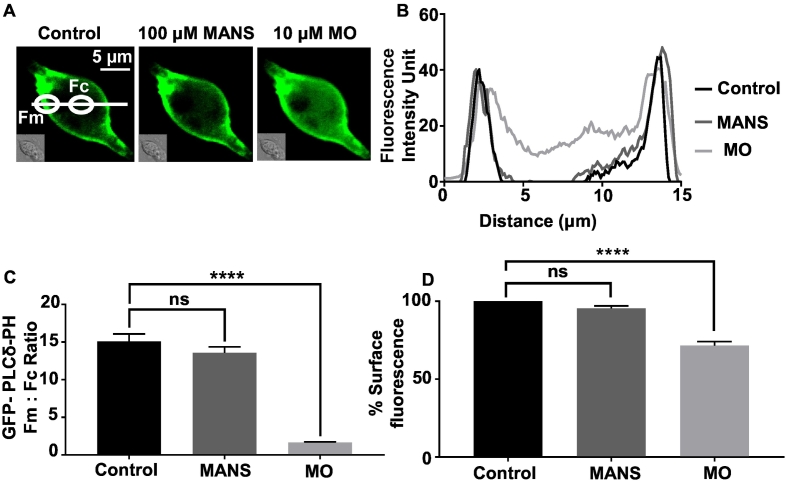
Effect of MANS and methoxamine (MO) on GFP-PLCδ-PH signals in single rat mesenteric artery vascular smooth muscle cells. A, Representative image from a single cell showing that in control conditions, the location of GFP-PLCδ-PH-mediated signals was predominantly expressed at the plasma membrane. In the same cell, application of MANS had no significant effect on PLCδ-PH-mediated signals while subsequent treatment with MO induced translocation of signals to the cytosol. B, Line scans showing GFP-PLCδ-PH signals across the cell width in control conditions, following treatment with MANS and subsequent application of MO. Mean data showing GFP-PLCδ-PH Fm:Fc ratios (C) and % surface fluorescence (D) in control conditions, treatment with MANS, followed by application of MO. Data from *n* = 6 animals, with *N* ≥ 4 cells per animal. Paired students *t*-test. ^⁎⁎⁎⁎^*P* < .001. ns indicates not significant.

**Fig. 7:**
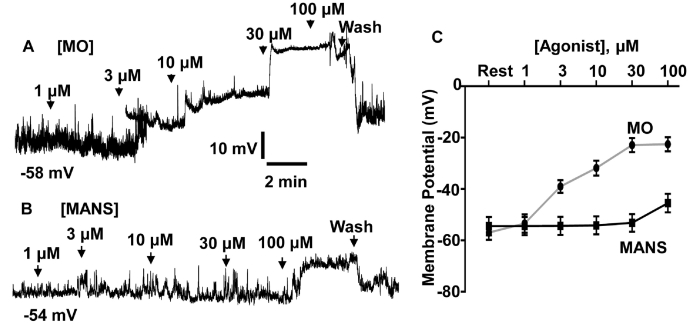
Effect of MANS and MO on membrane potential of rat mesenteric artery VSMCs. A and B, representative traces from two different VSMCs with resting membrane potentials of −58 mV and − 54 mV respectively. MO evoked concentration-dependent depolarisations between 1 and 100 μM whereas MANS induced a membrane depolarisation only at 100 μM. C, Mean data of the effect of MO and MANS membrane potential. Data from *N* = at least 6 patches from *n* = 3 animals.

Fig. 6 shows that in unstimulated VSMCs, GFP-PLCδ-PH signals were predominantly located at the plasma membrane with a mean Fm:Fc ratio of about 15, as expected when PIP_2_ is mainly located at the plasma membrane and there is limited cytosolic IP_3_. Fig. 6 illustrates that bath application of 100 μM MANS for 10 min failed to alter this signal distribution, whereas 10 μM MO induced translocation of GFP-PLCδ-PH signals to the cytosol that resulted in reduction of the mean Fm:Fc ratio by over 90%. These MO-induced signal changes are likely to represent PLC-mediated PIP_2_ hydrolysis at the plasma membrane and subsequent generation of cytosolic IP_3_ as previously described [40,41]. In support of these data, Fig. S8 shows that MO but not MANS altered PIP_2_ levels at the plasma membrane measuring using the PIP_2_-specific reporter GFP-tubby [46], as expected if MO induced PLC activity and MANS did not. Moreover, Fig. S9 shows that pre-treatment of mouse mesenteric artery segments with MANS did not significantly increase IP_3_ levels measuring with an Elisa assay, whereas pre-treatment with MO induced about a 5-fold increase in IP_3_ which is consistent with MO stimulating PLC activity. These results suggest that unlike MO, MANS is unlikely to produce significant effects on total PIP_2_ levels at the plasma membrane or increase PLC activity,

Another possibility is that MANS induces contraction through producing membrane depolarisation which leads to stimulation of VGCCs and Ca^2+^ influx [1,2]. We investigated this idea by comparing the effects of MANS and MO on membrane potential using whole-cell patch clamp recording under current-clamp conditions. Figs. 7A and C show that in the presence of the bath and patch pipette solution conditions used (see Methods) VSMCs had a resting membrane potential of about −55 mV, and that bath application of MO induced a concentration-dependent membrane depolarisation with a maximum effect of over 30 mV at above 30 μM. In contrast, Fig. 7B and C show that bath application of 1–30 μM MANS failed to induce a change in membrane potential whereas 100 μM MANS evoked a small depolarisation of less than 10 mV. These results suggest that distinct from MO, MANS is unlikely to produce a significant effect on membrane potential in VSMCs.

### 3.6 MARCKS and CaV1.2 subunits are co-localised in VSMCs

The above data suggests, it is unlikely that MANS induces contraction via increasing PLC activity or evoking membrane depolarisation. We therefore examined if MARCKS may directly modulate VGCC activity. We addressed this idea by investigating whether MANS and MO modulate interactions between MARCKS and CaV1.2 subunits, which are proposed to be the predominant VGCC subtype involved in producing vascular contractility [[42], [43], [44], [45]] and are likely to be involved in MANS-induced contractility (Figs. 5 and S7).

**Fig. 8:**
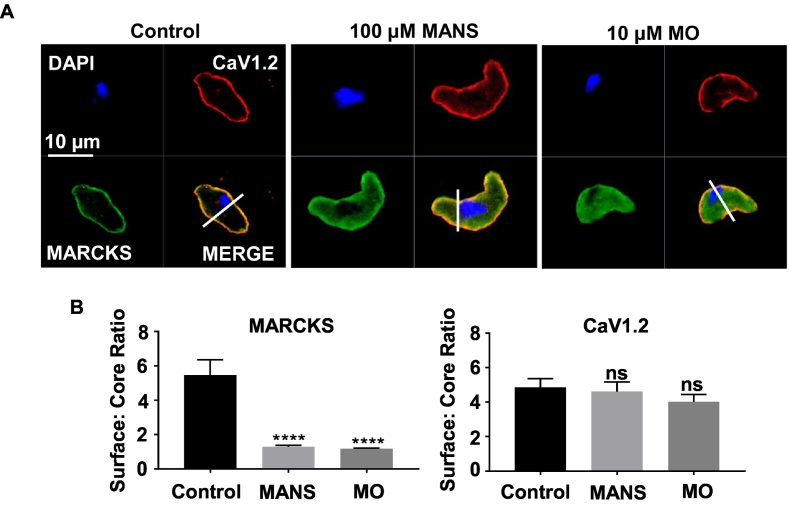
Cellular distribution of MARCKS and CaV1.2 in mouse mesenteric artery VSMCs. A and B, Representative images and mean data showing in that MARCKS (green) and CaV1.2 (red) co-localised at the plasma membrane of control VSMCs. Pre-treatment of two different VSMCs with either MANS or MO caused translocation of MARCKS to the cytosol whilst CaV1.2 remained at the plasma membrane. Data from *n* *=* 3 animals, with *N* ≥ 6 cells per animal. One-way ANOVA. ns indicates not significant. ^⁎⁎⁎⁎^*P* < .001. (For interpretation of the references to colour in this figure legend, the reader is referred to the web version of this article.)

Fig. 8 show that immunocytochemical staining for MARCKS and CaV1.2 were mainly located at, or close to, the plasma membrane of mouse mesenteric artery VSMCs in unstimulated cells, and that there was substantial co-localisation between these signals. Bath application of 100 μM MANS and 10 μM MO reduced expression of MARCKS near the plasma membrane which was accompanied by a noticeable increase in MARCKS expression within the cytosol. In contrast, MANS and MO failed to affect the expression distribution of CaV1.2. Furthermore, Fig. S10 shows that proximity ligation assays (PLA) produced robust puncta formation between MARCKS and CaV1.2 at, or close to, the plasma membrane of unstimulated mouse mesenteric artery VSMCs, which was reduced by over 70% following pre-treatment with 100 μM MANS and 10 μM MO.

These results suggest that MARCKS and CaV1.2 interact with each other in unstimulated VSMCs, and that these interactions are reduced by MANS and MO. Importantly, our data also suggest that reductions in MARCKS-CaV1.2 interactions by MANS and MO are associated with translocation of MARCKS from the plasma membrane to the cytosol.

### 3.7 MANS and MO alter interactions between PIP_2_, MARCKS, and CaV1.2

**Fig. 9:**
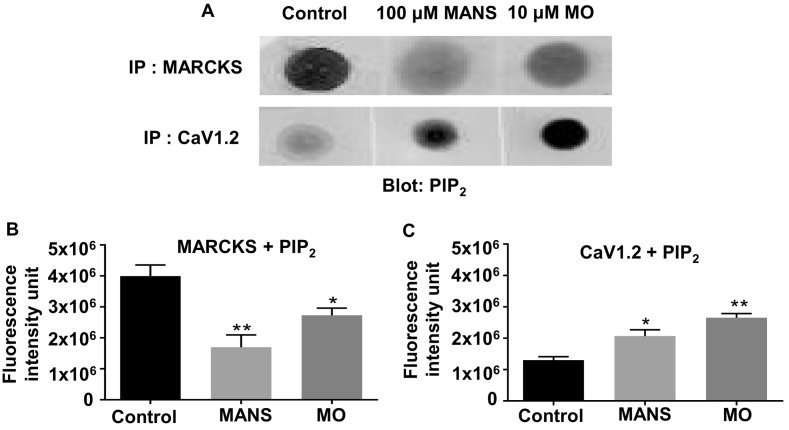
Interactions between PIP_2_ and MARCKS, and PIP_2_ and CaV1.2 in tissue lysates from rat mesenteric arteries. A, Representative dot-blot with an anti-PIP_2_ antibody after immunoprecipitation (IP) with either anti-MARCKS (top panels) or anti-CaV1.2 antibodies (bottom panels) from vessel segments pre-treated with distilled water (control), MANS, and MO. B and C, Mean data showing the effect of MANS or MO on PIP_2_ + MARCKS and PIP_2_ + CaV1.2 interactions. Data from *N* = 3 experimental preparations with *n* = 3 animals used per preparation. One-way ANOVA. ^⁎^*P* < .05, ^⁎⁎^*P* < .01.

Since MARCKS is a well-established plasma membrane PIP_2_-binding protein or PIPmodulin [11], we investigated if the reduction in MARCKS-CaV1.2 interactions and translocation of MARCKS into the cytosol produced by MANS and MO were accompanied by changes in PIP_2_ associated with these two molecules. Using immunoprecipitation and dot-blot methods as previously described [30], Fig. 9 shows that there was a greater signal for PIP_2_ interactions with MARCKS than for PIP_2_ with CaV1.2 in unstimulated rat mesenteric artery tissue lysates. Following pre-treatment of vessels with 100 μM MANS and 10 μM MO the strength of these signals was reversed, with greater binding observed between of PIP_2_ and CaV1.2 than for PIP_2_ and MARCKS.

### 3.8 MANS increase VGCC activity through a PIP_2_-dependent mechanism

Our findings suggest that within MARCKS-CaV1.2 complexes, PIP_2_ may be predominantly bound to MARCKS and not CaV1.2. However, upon stimulation with MANS and MO, MARCKS is translocated from the plasma membrane to the cytosol leading to release of PIP_2_ that binds to CaV1.2. This suggests that inhibition of MARCKS induces vascular contractility by increasing VGCC activity through a PIP_2_-dependent mechanism. We explored this idea by studying the effect of MANS on VGCC current activity using Ba^2+^ as the charge carrier in rat mesenteric artery VSMCs using whole-cell patch clamp recording under voltage-clamp conditions.

**Fig. 10:**
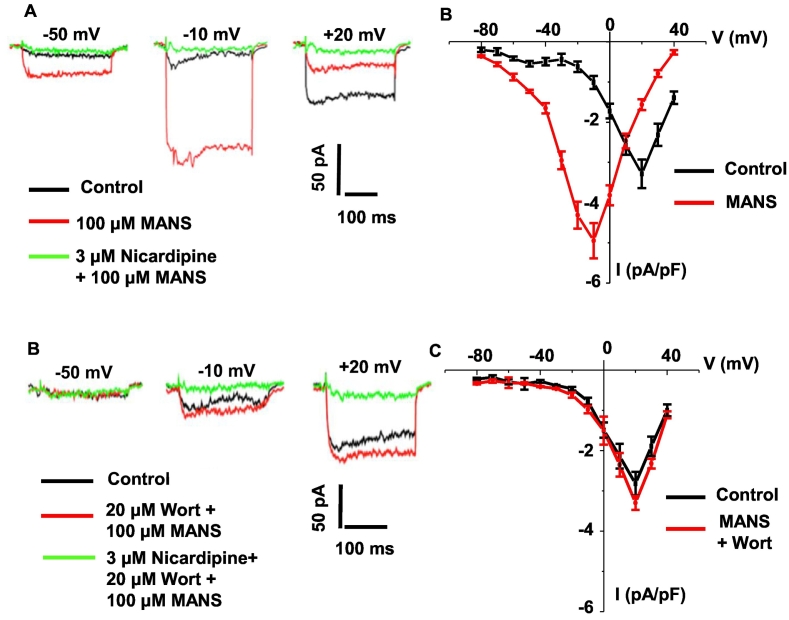
Effect of MANS on whole-cell VGCC activity in single rat mesenteric artery VSMCs. A, Representative traces showing that control whole-cell VGCC currents (black) from freshly isolated rat mesenteric artery VSMCs were significantly increased at −50 mV and − 10 mV but reduced at +20 mV following bath application of MANS (red) and that VGCC currents were subsequently blocked by nicardipine (green). B, Mean current-voltage (I/V) relationship of VGCC currents showing that MANS (red) produced a significant increase in peak amplitude and a negative shift in the mean activation threshold. C and D, Representative traces and mean I/V relationship of VGCC currents showing that pre-treatment of VSMCs with wortmannin (wort) attenuated the excitatory effects of MANS (red). Data from *n* = 6 animals, with *N* ≥ 3 patches per animal. (For interpretation of the references to colour in this figure legend, the reader is referred to the web version of this article.)

Fig. 10A show that applying 300 ms voltage pulses from −80 mV to +40 mV in 10 mV steps from a holding potential of −60 mV induced whole-cell inward currents which activated at about −60 mV, reached a peak amplitude at about +20 mV, and were inhibited by the VGCC blocker nicardipine. These characteristics are consistent with activation of whole-cell VGCC currents as previously described in VSMCs [[47], [48], [49], [50]]. Bath application of 100 μM MANS produced a pronounced increase in nicardipine-sensitive whole-cell inward currents, shifting the mean activation curve to more negative membrane potentials and increasing mean peak amplitude by over 50%. Moreover, Fig. 10B show that pre-treatment of VSMCs with a high concentration of wortmannin (20 μM), a PI4/PI5 kinase inhibitor that leads to depletion of PIP_2_ levels [51,52], did not affect the activation curve of whole-cell inward currents but did prevent MANS-induced negative shift in the mean activation curve and increase in mean peak amplitude. This suggests that PIP_2_ is likely to mediate the excitatory effect of MANS on VGCC activity.

To provide evidence that wortmannin reduces PIP_2_ levels, Fig. S11 shows that wortmannin reduced the plasma membrane signals of the highly selective PIP_2_ biosensor GFP-Tubby and the PIP_2_/IP_3_ biosensor GFP-PLCδ-PH in rat mesenteric artery VSMCs. Moreover, Fig. S11 shows that total PIP_2_ levels from tissue lysates of mouse mesenteric artery measured using dot-blot analysis was reduced by pre-treatment with wortmannin.

## 4 Discussion

The present study provides the first evidence that the PIP_2_-binding protein MARCKS regulates vascular contractility and reveals its potentially important role in mediating vasoconstrictor-induced contractions. Our initial findings suggest that MARCKS regulates contraction by modulating the activity of VGCCs by PIP_2_. These results identify novel cellular mechanisms involved in regulating vascular contractility, which are likely to have important consequences for future understanding of physiological and pathological vascular function.

### 4.1 MARCKS regulates vascular contractility

We show that MARCKS is expressed in mouse and rat mesenteric artery VSMCs, where it is predominantly distributed at, or close to, the plasma membrane. This is consistent with earlier studies from ferret portal vein [26], human coronary artery [[27], [28], [29]] and rabbit and mouse portal vein [30].

We used a well-established pharmacological intervention to investigate the role of MARCKS in vascular contractility. The selective MARCKS inhibitor, MANS, evoked robust, sustained and reproducible vascular contractions in mouse mesenteric arteries, which were equivalent to contractions induced by stimulation of α_1_-adrenoceptors by methoxamine (MO) and thromboxane receptors by U46619. MANS is a selective inhibitory peptide, which corresponds to the myristoylated N-terminal region that anchors MARCKS at the plasma membrane and has been extensively used to investigate the function of MARCKS in many different preparations [[33], [34], [35], [36]]. MANS is used at relatively high concentrations (up to 100 μM) as it acts by competing for endogenous MARCKS at the plasma membrane and MARCKS is thought to have a cellular concentration of about 10 μM (similar to the cellular concentration of PIP_2_) [33,34]. Thus, MANS is used at 10-fold greater concentrations than endogenous MARCKS to produce sufficient inhibition.

To provide molecular evidence that the effects of MANS were not produced through off-target actions, we showed that reducing MARCKS expression levels and, distribution at, or close to, the plasma membrane with MARCKS-targeted morpholino oligonucleotides greatly inhibited MANS-evoked contractions. Contractions evoked by MANS and MO in vessels pre-treated with scrambled morpholino oligomers had mean EC_50_ and E_max_ values similar to values recorded in freshly isolated arteries, and MARCKS-targeted oligomers did not alter expression of α-tubulin or the expression and cellular distribution of CaV1.2 proteins. These results suggest that the transfection process is unlikely to alter vasoconstrictor-mediated responses or involvement of MARCKS inferred through use of MANS, and importantly that MARCKS-targeted oligomers have selectivity against MARCKS. All these findings increase the validity to our approach. The lack of an effect of MARCKS-targeted oligomers on expression levels and cellular distribution of CaV1.2 is also of importance as we show that CaV1.2 is likely to be involved in MARCKS-evoked contractions (see below).

A significant result was that contractions induced by MO and U46619 were also substantially inhibited by MARCKS-targeted oligomers, with changes in EC_50_ and E_max_ values equivalent to those observed with MANS-evoked contractions. These striking findings pose an interesting conflict; why does pharmacological inhibition of MARCKS produce contractility whereas knockdown of MARCKS expression reduces vasoconstrictor-evoked contractility? These seemingly opposing data can be explained if MARCKS exerts an inhibitory effect on contractility in unstimulated vessels and that disinhibition of this action of MARCKS is required for MANS- and vasoconstrictor-mediated contractility. As such, disinhibition of this MARCKS inhibitory action in unstimulated vessels by acute application or MANS or vasoconstrictor agents (e.g. MO and U46619) induce contraction. However, following knockdown of MARCKS, MANS and vasoconstrictor-stimulated disinhibition of MARCKS is curtailed leading to a reduction in contraction. These ideas suggest is that disinhibition of MARCKS causing contraction is unlikely to be a pharmacological phenomenon but is an important physiological pathway which is necessary for vasoconstrictor-mediated contractility.

It is possible that MARCKS-targeted oligomers may have reduced MANS- and vasoconstrictor-mediated contractions by having non-selective effects on the activity of VGCCs and/or by reducing the ability of vessels to contract. However, this seems unlikely, contractions induced by KCl and ionomyocin, which induce contractility through stimulating VGCCs and providing direct Ca^2+^ influx to activate Ca^2+^-dependent contractile mechanisms respectively were similar in vessels transfected with scrambled and MARCKS-targeted oligomers. It might have been expected that knockdown of MARCKS would alter resting tension, produced in the normalisation process to represent a physiological blood pressure of 100 mmHg, but our results showed that resting tension was not different between vessels pre-treated with scrambled and MARCKS-targeted oligomers. This should perhaps be investigated in future experiments using pressure myography which may provide greater resolution.

In conclusion, these findings indicate that endogenous MARCKS has a pronounced inhibitory action on vascular contractility which can be modulated by direct inhibition of MARCKS and vasoconstrictor stimulation. MARCKS has previously been shown to regulate proliferation and migration of VSMCs and has been implicated in the progression of intima hyperplasia [[27], [28], [29]]. However, this is first time that MARCKS has been implicated in regulating contractility.

### 4.2 MANS evokes vascular contractility via activation of VGCCs

It is well-established that Ca^2+^ influx through activation of VGCCs plays a central role in mediated vascular contraction involving two VGCC subtypes, L-type (CaV1.2) and T-type (CaV3.1/3.2), with L-Type VGCCs considered to have the predominant role in initiating vasoconstrictor-mediated contraction [[42], [43], [44], [45]]. Our results show that MANS-evoked contractions were inhibited by several proposed selective L-type and T-type VGCC blockers, with each agent able to produce complete relaxation. The IC_50_ values for the blockers against MANS-evoked contractions were relatively high compared to known values for these channel subtypes [[53], [54], [55], [56], [57]]. This may be due to the blockers being applied to pre-contracted vessels and not pre-incubated before contraction was induced and/or that multiple VGCC subtypes are involved. It is therefore difficult to accurately determine from these experiments if either or both L-type and T-type VGCCs are involved in mediating MANS-evoked contractions. A potential discrimination is provided by the effect of Ni^2+^, reported to offer T-type VGCCs selectivity at concentrations less than 50 μM [55], which blocked MANS-evoked contractions with an IC_50_ of 250 μM suggesting a predominant role for L-type VGCC subtype. What is certain is that activation of VGCCs play a central role in the pathway whereby disinhibition of MARCKS by MANS induces contraction.

### 4.3 MARCKS regulates interactions between VGCCs and PIP_2_

It is recognised that MANS, by competing with MARCKS at the plasma membrane, induces translocation of MARCKS from the plasma membrane to the cytosol that reduces electrostatic interactions between MARCKS and PIP_2_ causing release of PIP_2_ into the local environment [33,34]. We therefore considered that MANS may induce VGCC-mediated contractions by inducing a rise in PIP_2_ levels, which acts as a substrate for PLC activity to induce contraction via the familiar phosphatidylinositol transduction pathway. In addition, MANS may also induce VGCCs and contraction by evoking a membrane depolarisation. However, MANS failed to alter the distribution of the PIP_2_/IP_3_ biosensor GFP-PLCδ-PH and PIP_2_-specific reporter GFP-tubby and had little effect on membrane potential in VSMCs. This contrasts with stimulation of α_1_-adrenoceptors, which induced a translocation of GFP-PLCδ-PH and GFP-tubby from the plasma membrane to the cytosol in VSMCs that is indicative of PLC activity [40,41], and induced a significant membrane depolarisation. These findings are further supported by previous evidence indicating that sequestered PIP_2_ by MARCKS does not interfere with PLC activity [12,24].

We next focused on the possibility that MANS and MO induce contraction through regulating interactions between MARCKS, VGCCs, and PIP_2_. We studied the L-type CaV1.2 subunit as this is considered the dominant VGCC involved in initiating vascular contractility by MANS from the pharmacological profile [[42], [43], [44], [45]]. Using immunocytochemistry and PLA, we clearly show that MARCKS-CaV1.2 interactions are present in unstimulated VSMCs and that these associations occur at, or close to, the plasma membrane. In addition, MANS and MO both cause dissociation of MARCKS-CaV1.2 interactions and MARCKS to translocate the cytosol. Moreover, we show that in unstimulated vessel segments PIP_2_ was bound more to MARCKS than CaV1.2, but that this binding profile was reversed following pre-treatment with MANS and MO. These results are similar to earlier studies showing that the known inhibitors of MARCKS, CaM and PKC [[15], [16], [17], [18], [19], [20], [21], [22], [23], [24], [25], [26],^30^], and MO [30] lead to translocation of MARCKS from the plasma membrane to the cytosol, and that MO induces preferential changes in PIP_2_ binding at MARCKS-TRPC1 interactions [30]. These findings provide further evidence that MANS induces vascular contractility by causing disinhibition of an endogenous MARCKS inhibitory pathway. Moreover, PIP_2_ imaging with GFP-PLCδ-PH, GFP-tubby and dot-blots, indicate that redistribution of PIP_2_ from MARCKS to CaV1.2 subunits and not changes in total PIP_2_ levels may be an important step in this pathway.

To provide further context to our ideas that MARCKS regulates VGCCs via a PIP_2_-dependent mechanism, MANS induced an increase in whole-cell VGCC currents in VSMCs through shifting the activation curve to more negative membrane potentials and augmenting mean peak amplitude. These MANS-mediated increases in VGCC currents were prevented by pre-treatment of VSMCs with wortmannin which depletes endogenous PIP_2_ levels. This is consistent with studies showing that PIP_2_ facilitates L-, T-, and P-type VGCC activity in overexpression systems [[3], [4], [5], [6], [7], [8], [9], [10]]. High concentration of wortmannin (20 μM) depletes PIP_2_ levels through inhibiting PI-4/PI-5 kinase-mediated PIP_2_ synthesis (see Fig. S10) [4]. However, it should be noted that high concentrations of wortmannin is also likely to inhibit myosin light chain kinase (MLCK) and PI-3 kinase, and therefore due caution should be given to these results.

Taken together, the present work indicates that MARCKS regulates vascular contractility by modulating VGCC activity (see Fig. S12). In unstimulated VSMCs, MARCKS forms interactions with CaV1.2 and acts as a PIP_2_ buffer or PIPmodulin [11] to sequester local PIP_2_ levels that reduces PIP_2_-mediated facilitation of VGCC activity. Disinhibition of MARCKS by MANS leads to dissociation of MARCKS-CaV1.2 interactions and translocation of MARCKS to the cytosol, which releases sequestered PIP_2_ at the plasma membrane where it binds to and facilitates VGCC activity to promote contraction. In the future it will be important to identify if both L-type (CaV1.2) and T-type (CaV3.1/3.2) VGCC subtypes are involved, and whether pore-forming α subunits and auxiliary subunits such as β and α_2_δ contribute to these responses. Moreover, a detailed examination of exogenous PIP_2_ and endogenous PIP_2_ actions on VGCC activity is required using respectively: water soluble forms of PIP_2_ such as diC8-PIP_2_ and established techniques to deplete endogenous PIP_2_ levels such as *Danio rerio* voltage-sensing phosphatase (DrVSP) and rapamycin-FRB/FKBP-5′ phosphatase is required [58]. In the longer term it will be important to reveal the structure of PIP2-VGCC interaction sites.

### 4.4 Future implications for understanding cellular mechanisms regulating vascular contractility

Our findings reveal that α_1_-adrenoceptor stimulation produced similar actions to MANS on MARCKS-CaV1.2 interactions, MARCKS translocation, and changes in PIP_2_ binding to MARCKS and CaV1.2 (Fig. S12). In contrast, stimulation of α_1_-adrenoceptors evoked a substantial membrane depolarisation of VSMCs whereas MANS had little effect on membrane potential. It is generally considered that stimulation of Gq-protein receptor-mediated pathways by vasoconstrictors induces contractility through inducing membrane potential depolarisation through modulation of ion channels such as cation, Cl^−^, and K^+^ channels which cause activation of VGCCs and Ca^2+^ influx [1,2,59]. The present study poses important questions about these established vasoconstrictor-mediated pathways by suggesting that, in addition to membrane depolarisation, these Gq-protein receptor-mediated pathways may also cause disinhibition of MARCKS to directly activate of VGGCs to produce contraction. Essentially, VGCCs become receptor-operated channels at the resting membrane potential through the facilitatory effect of PIP_2_ released from MARCKS, which shifts the activation threshold of VGCCs to more negative membrane potentials. The idea that VGCCs may be receptor-operated channels and are activated independently of membrane depolarisation is not new, some 30 years ago, Nelson and colleagues presented evidence that vasoconstrictors activate VGCCs held at resting membrane potentials [60]. There is no doubt that this concept needs revisiting, such as does α_1_-adrenceptor-induced contractions require MARCKS and are known Gq-protein receptor-mediated CaM and/or PKC pathways coupled to disinhibition of MARCKS and regulation of contractility [26,30]. Whatever the outcome of these future experiments, the present study provides the first evidence that MARCKS has a critical role in regulating vascular contractility and offers a potential new target for modulating contractility in treating cardiovascular disease.

## Declaration of competing interest

None.

## Appendix A Supplementary data

Supplementary figures Image 1

Supplementary information Image 2
