# Supplementary material for: MARCKS mediates vascular contractility through regulating interactions between voltage-gated Ca2+ channels and PIP2
Source: Vascul Pharmacol. 2020 Sep;132:106776. doi: 10.1016/j.vph.2020.106776 (PMC7549404; doi:10.1016/j.vph.2020.106776)
Supplement: Supplementary file 1 — Supplementary figures [file mmc1.pptx]

## Slide 1
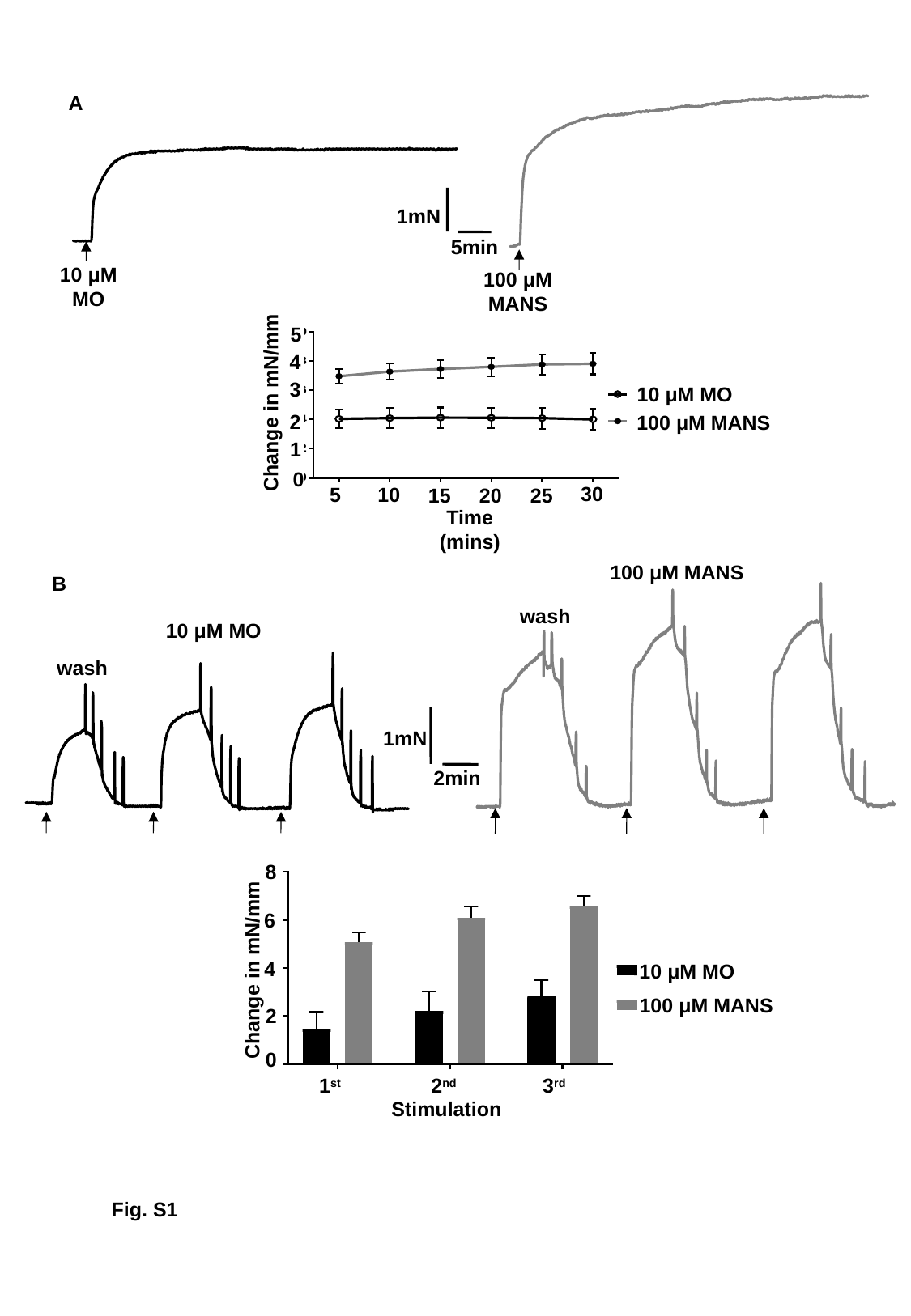

A
100 μM MANS
10 μM MO
1mN
5min
5
4
3
10 μM MO
Change in mN/mm
2
100 μM MANS
1
0
30
10
5
20
25
15
Time (mins)
100 μM MANS
B
wash
10 μM MO
wash
1mN
2min
8
6
4
Change in mN/mm
10 μM MO
100 μM MANS
2
0
3rd
2nd
1st
Stimulation
Fig. S1

## Slide 2
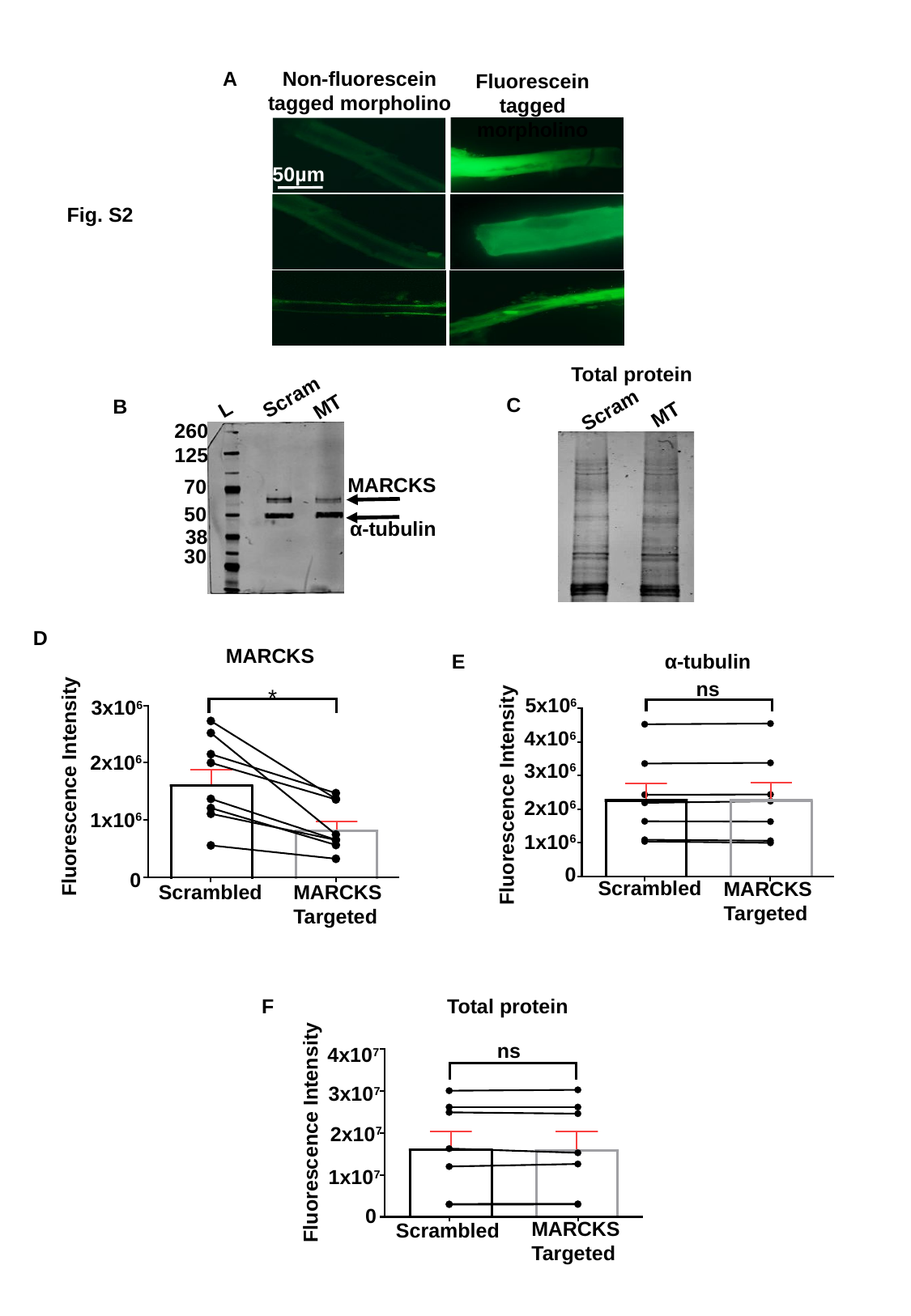

A
Non-fluorescein
tagged morpholino
Fluorescein
tagged morpholino
50µm
Fig. S2
Total protein
Scram
MT
Scram
MT
L
260
125
70
50
38
30
MARCKS
α-tubulin
C
B
D
MARCKS
3x10^6^
2x10^6^
Fluorescence Intensity
1x10^6^
0
MARCKS
Targeted
Scrambled
E
α-tubulin
4x10^6^
3x10^6^
Fluorescence Intensity
2x10^6^
1x10^6^
0
Scrambled
MARCKS
Targeted
5x10^6^
ns
F
Total protein
3x10^7^
2x10^7^
Fluorescence Intensity
1x10^7^
0
MARCKS
Targeted
Scrambled
4x10^7^
ns

## Slide 3
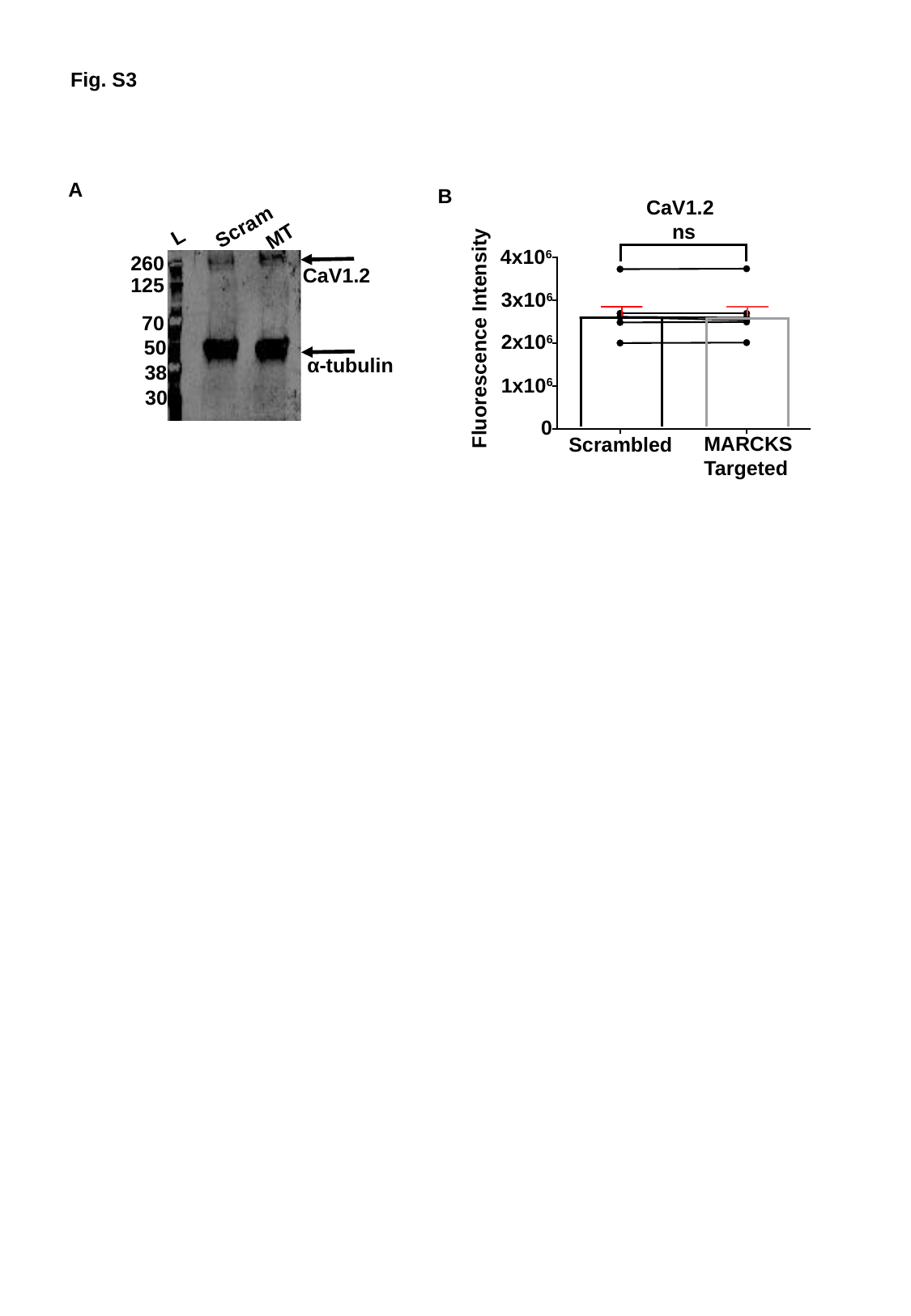

Fig. S3
A
B
CaV1.2
4x10^6^
3x10^6^
Fluorescence Intensity
2x10^6^
1x10^6^
0
MARCKS
Targeted
Scrambled
Scram
MT
L
260
125
70
50
38
30
CaV1.2
α-tubulin
ns

## Slide 4
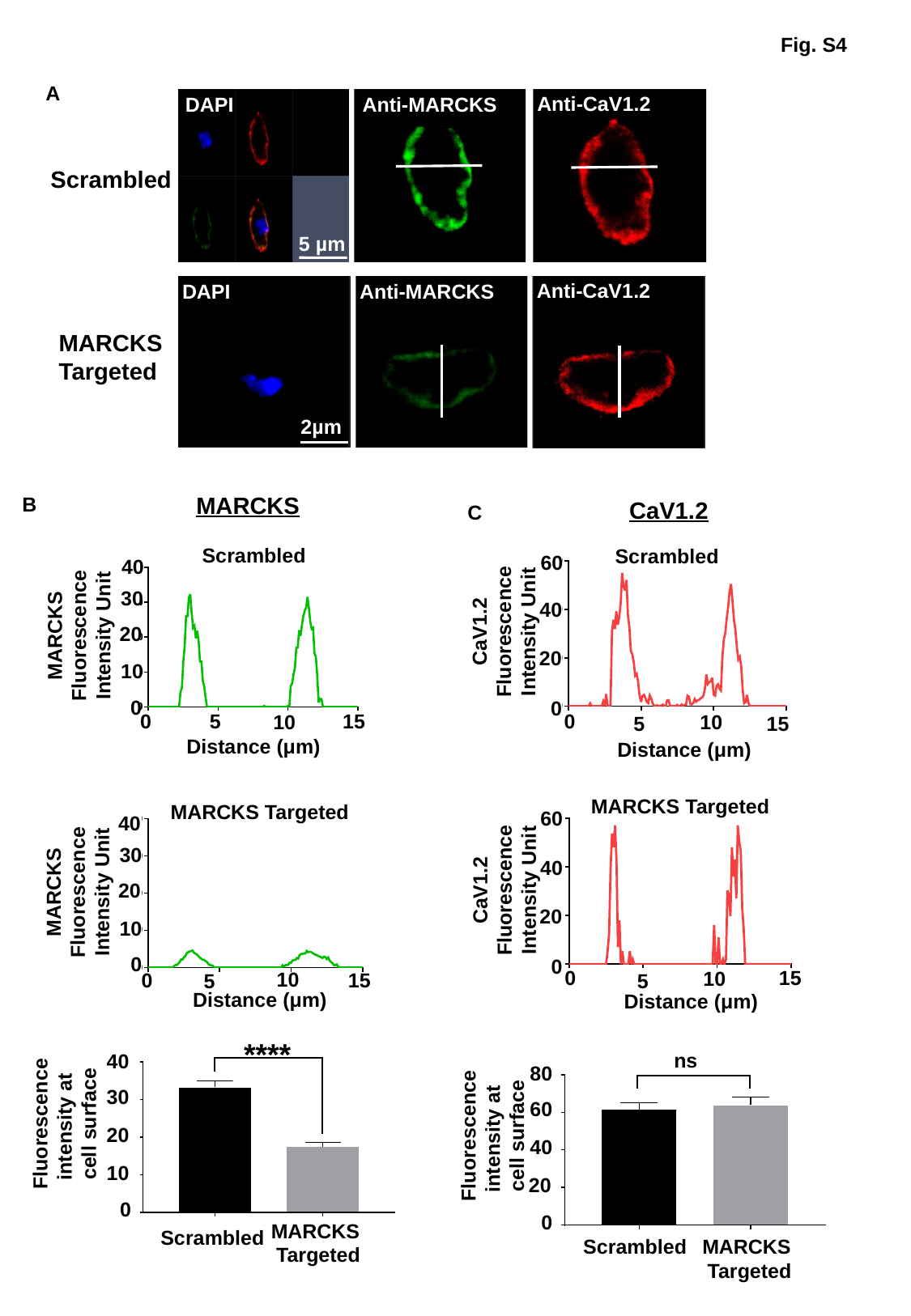

Fig. S4
A
Anti-CaV1.2
Anti-MARCKS
DAPI
Scrambled
5 µm
Anti-CaV1.2
DAPI
Anti-MARCKS
MARCKS
Targeted
2µm
MARCKS
B
CaV1.2
C
Scrambled
40
30
MARCKS
Fluorescence
Intensity Unit
20
10
0
15
5
0
10
Distance (μm)
Scrambled
60
CaV1.2
Fluorescence
Intensity Unit
40
20
0
0
10
15
5
Distance (μm)
MARCKS Targeted
60
CaV1.2
Fluorescence
Intensity Unit
40
20
0
15
0
10
5
Distance (μm)
MARCKS Targeted
40
30
MARCKS
Fluorescence
Intensity Unit
20
10
0
10
0
15
5
Distance (μm)
40
Fluorescence
intensity at
cell surface
30
20
10
0
Scrambled
MARCKS
Targeted
****
ns
80
Fluorescence
intensity at
cell surface
60
40
20
0
Scrambled
MARCKS
Targeted

## Slide 5
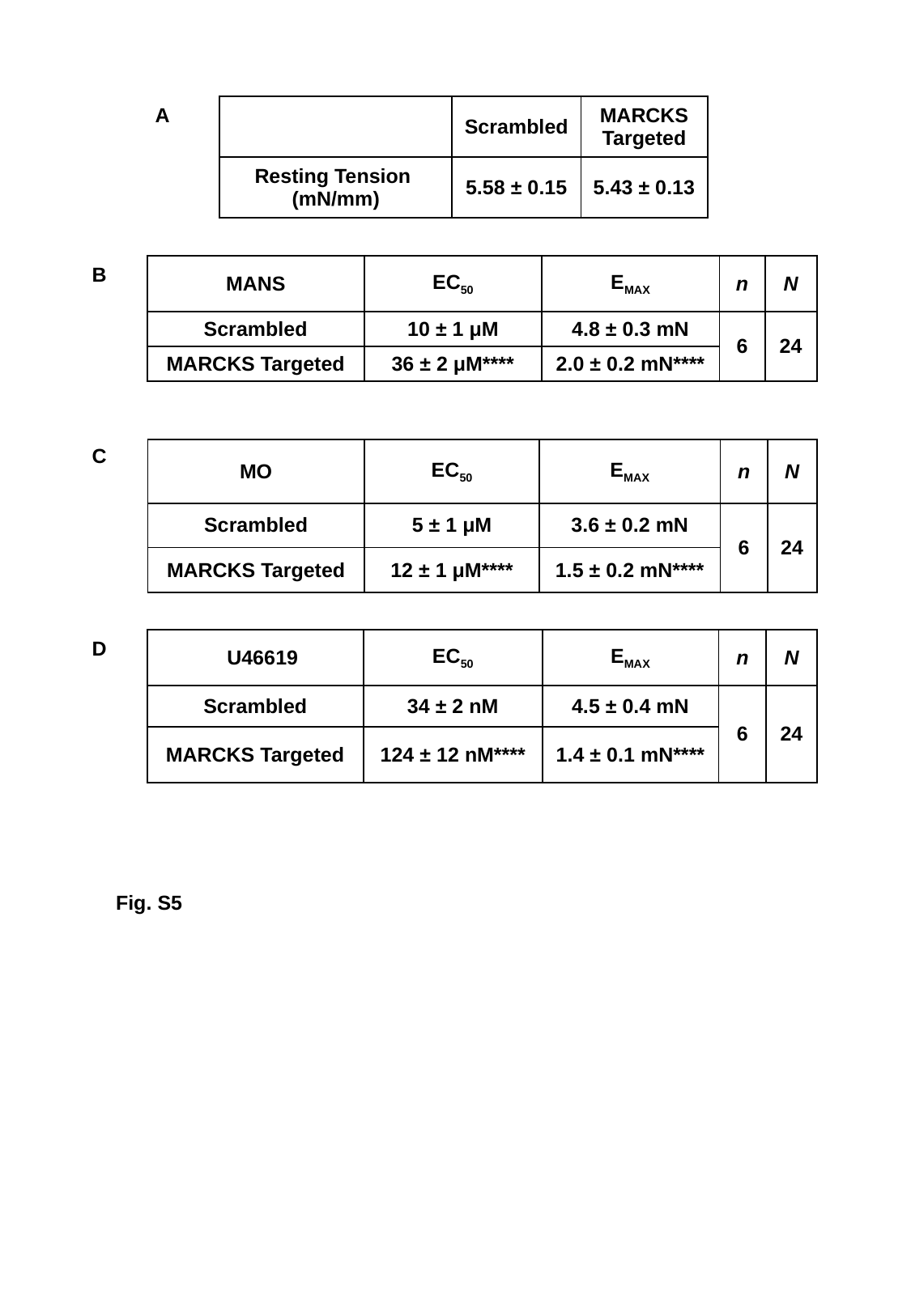

A
| | Scrambled | MARCKS Targeted |
| --- | --- | --- |
| Resting Tension (mN/mm) | 5.58 ± 0.15 | 5.43 ± 0.13 |
B
| MANS | EC50 | EMAX | n | N |
| --- | --- | --- | --- | --- |
| Scrambled | 10 ± 1 μM | 4.8 ± 0.3 mN | 6 | 24 |
| MARCKS Targeted | 36 ± 2 μM\*\*\*\* | 2.0 ± 0.2 mN\*\*\*\* | | |
C
| MO | EC50 | EMAX | n | N |
| --- | --- | --- | --- | --- |
| Scrambled | 5 ± 1 μM | 3.6 ± 0.2 mN | 6 | 24 |
| MARCKS Targeted | 12 ± 1 μM\*\*\*\* | 1.5 ± 0.2 mN\*\*\*\* | | |
D
| UU46619 | EC50 | EMAX | n | N |
| --- | --- | --- | --- | --- |
| Scrambled | 34 ± 2 nM | 4.5 ± 0.4 mN | 6 | 24 |
| MARCKS Targeted | 124 ± 12 nM\*\*\*\* | 1.4 ± 0.1 mN\*\*\*\* | | |
Fig. S5

## Slide 6
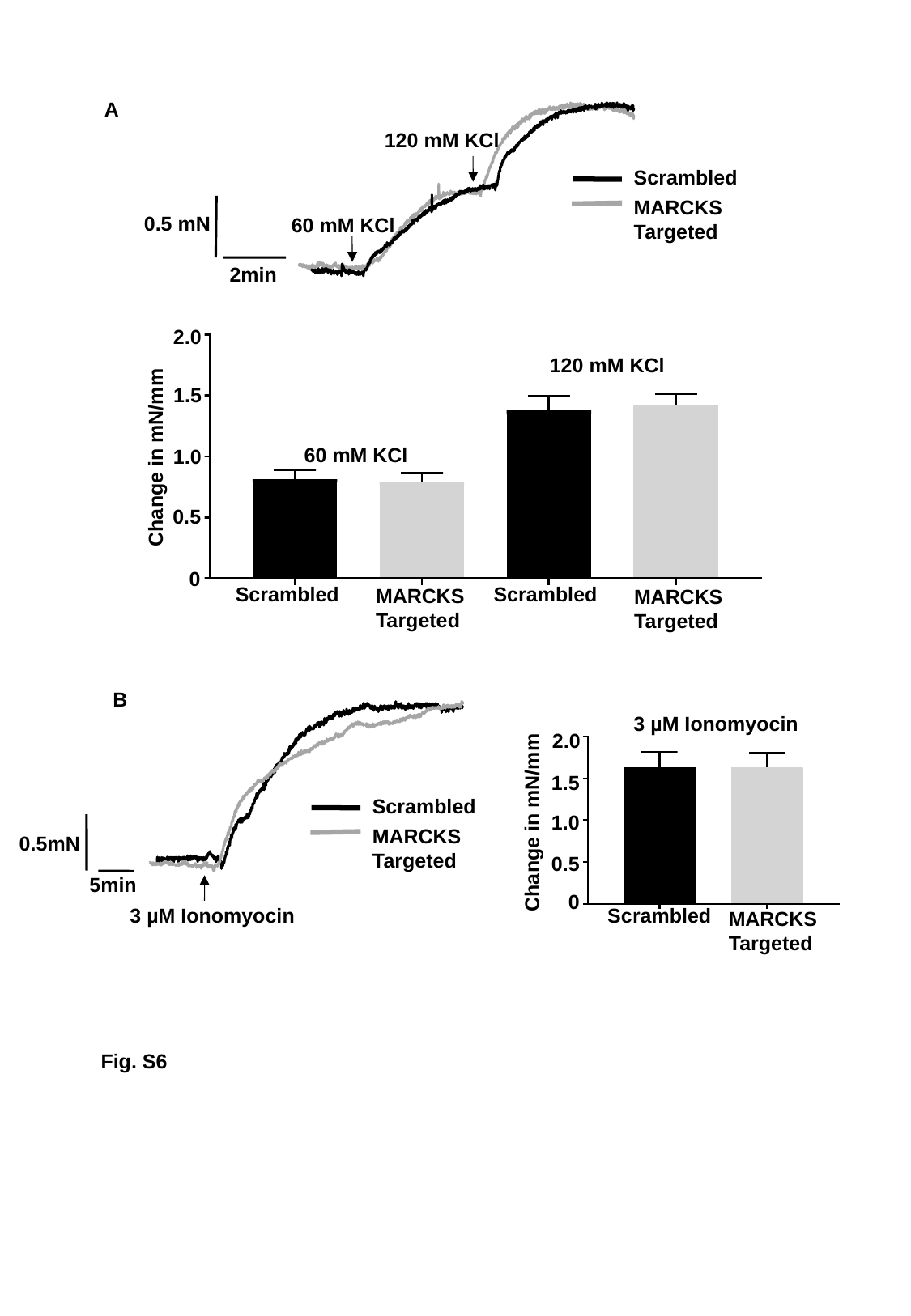

A
120 mM KCl
Scrambled
MARCKS
Targeted
60 mM KCl
0.5 mN
2min
2.0
120 mM KCl
1.5
60 mM KCl
1.0
Change in mN/mm
0.5
0
Scrambled
Scrambled
MARCKS
Targeted
MARCKS
Targeted
B
Scrambled
MARCKS
Targeted
0.5mN
5min
3 µM Ionomyocin
3 µM Ionomyocin
2.0
1.5
1.0
Change in mN/mm
0.5
0
Scrambled
MARCKS
Targeted
Fig. S6

## Slide 7
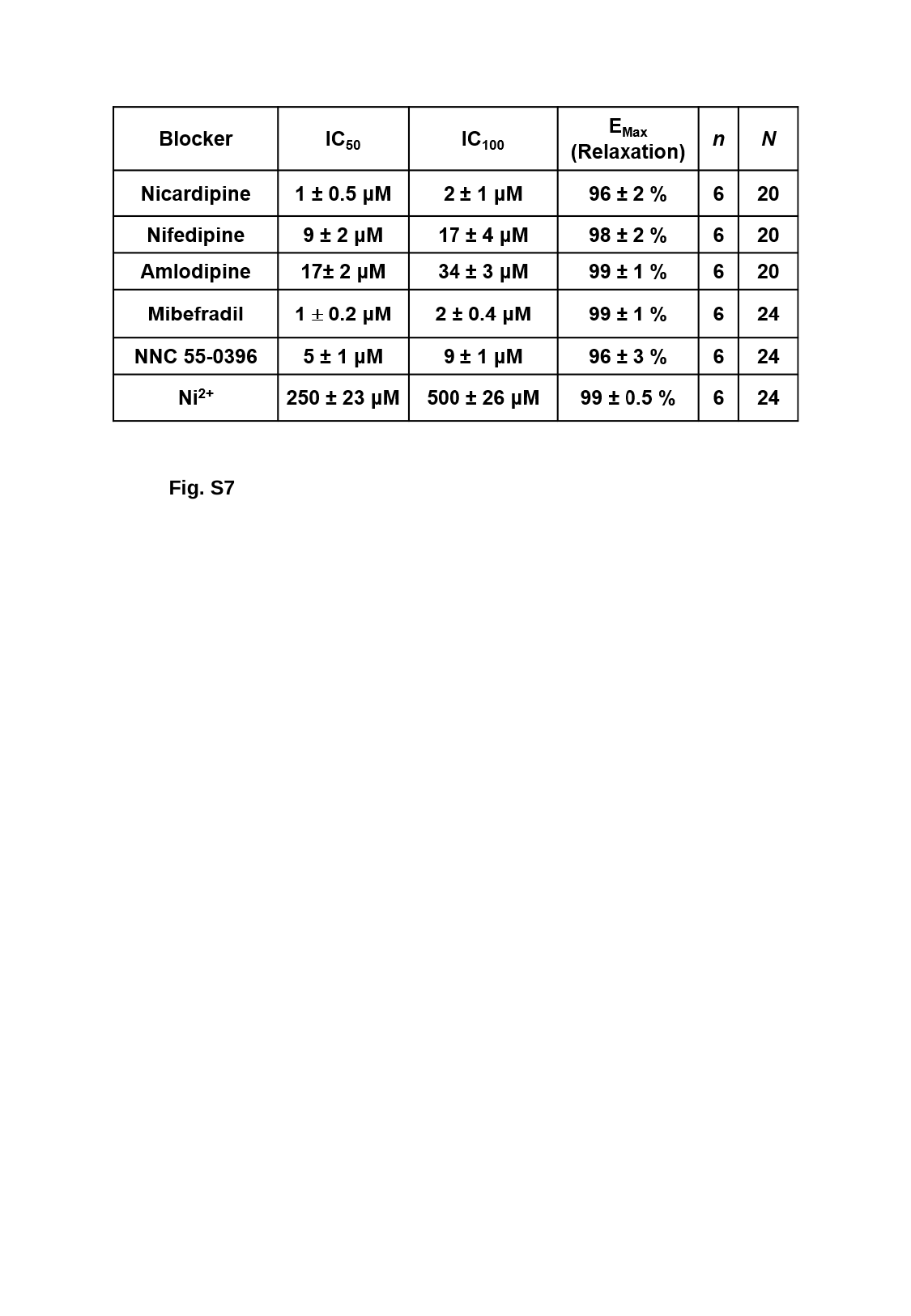

Fig. S7

## Slide 8
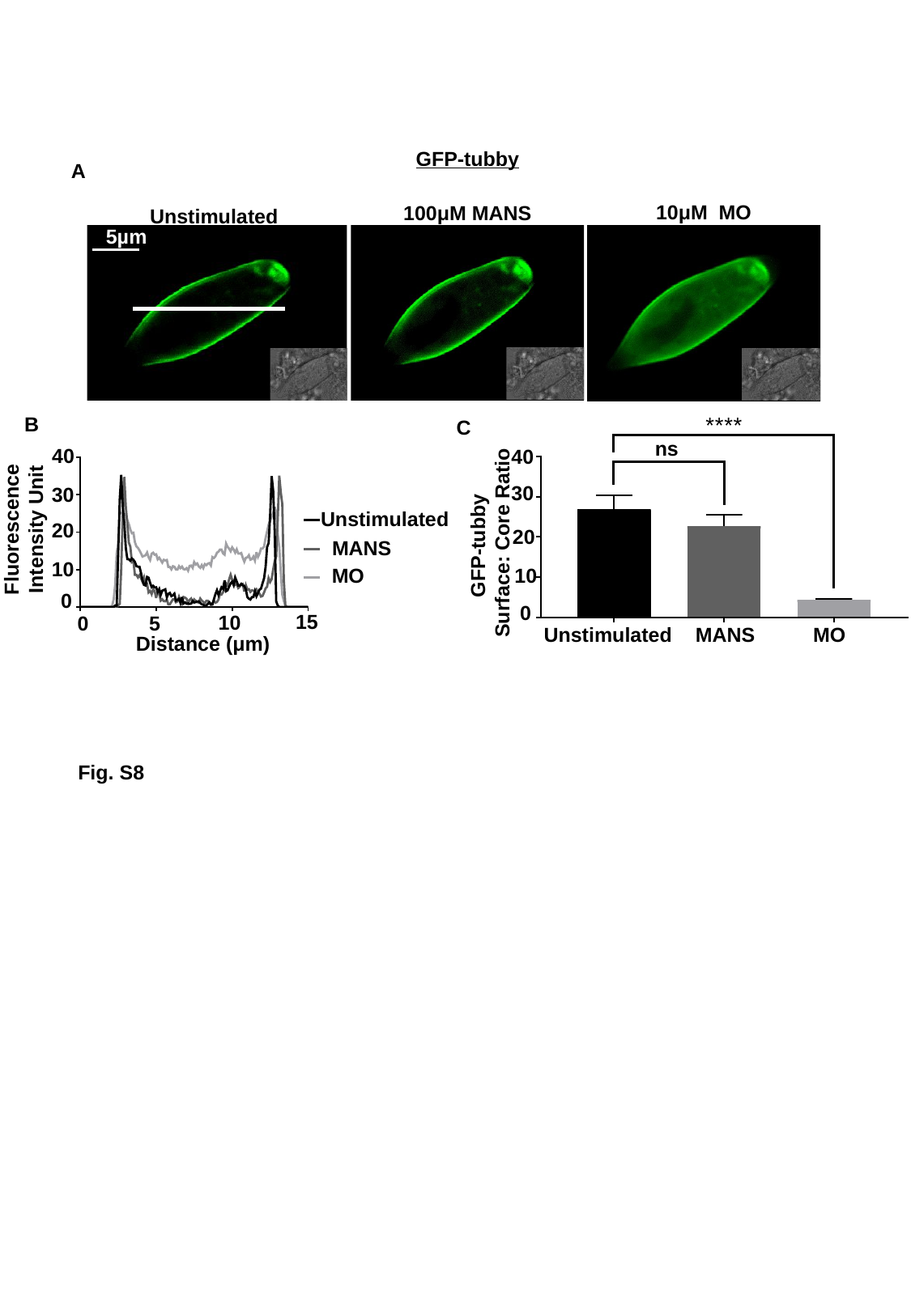

GFP-tubby
A
10μM MO
100μM MANS
Unstimulated
5µm
B
C
ns
40
30
GFP-tubby
Surface: Core Ratio
20
10
0
Unstimulated
MANS
MO
40
30
Unstimulated
20
MANS
10
 MO
0
15
10
5
0
Distance (μm)
Fluorescence
Intensity Unit
Fig. S8

## Slide 9
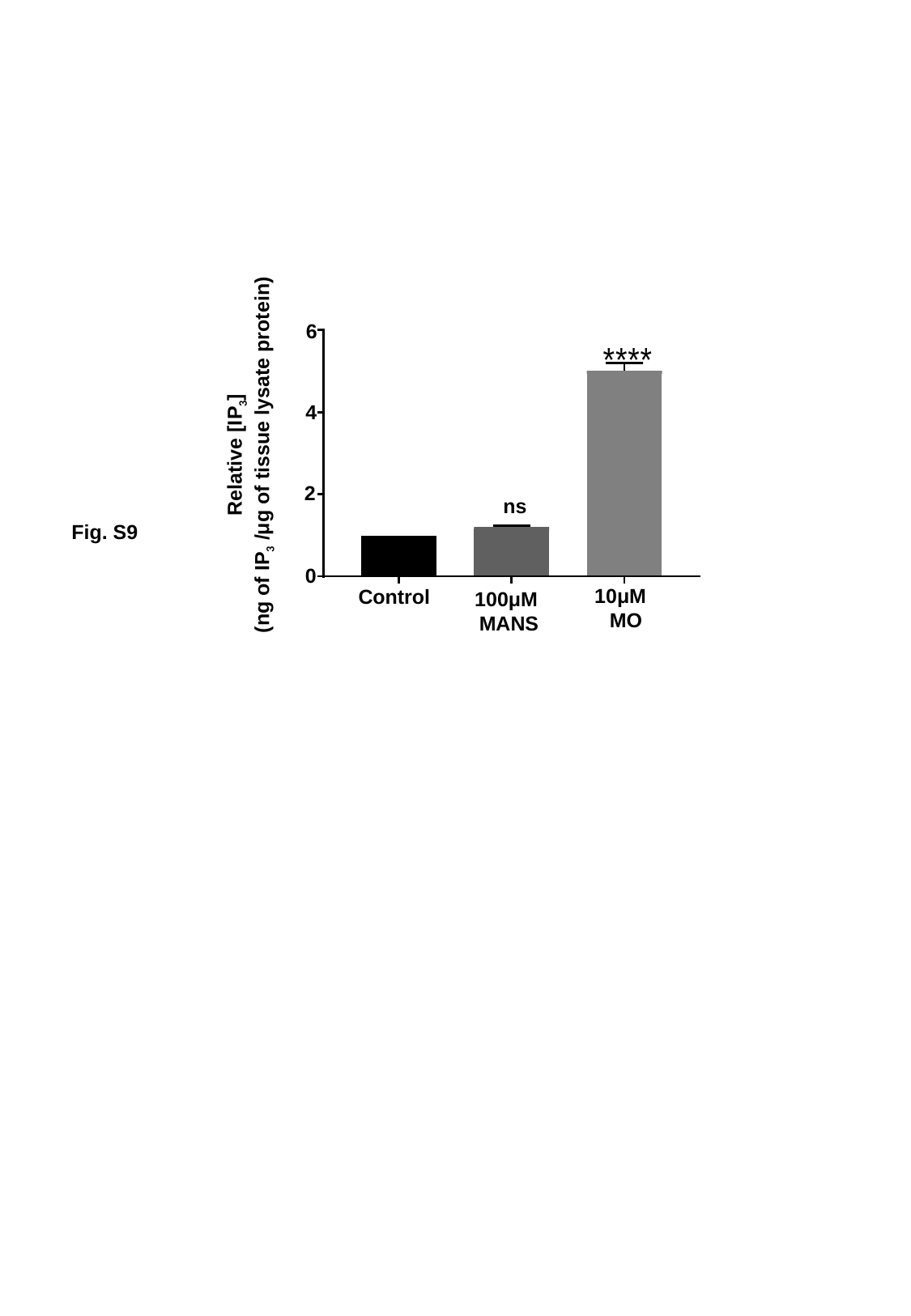

6
4
Relative [IP3]
(ng of IP3 /μg of tissue lysate protein)
2
0
10μM
MO
Control
100μM
MANS
ns
Fig. S9

## Slide 10
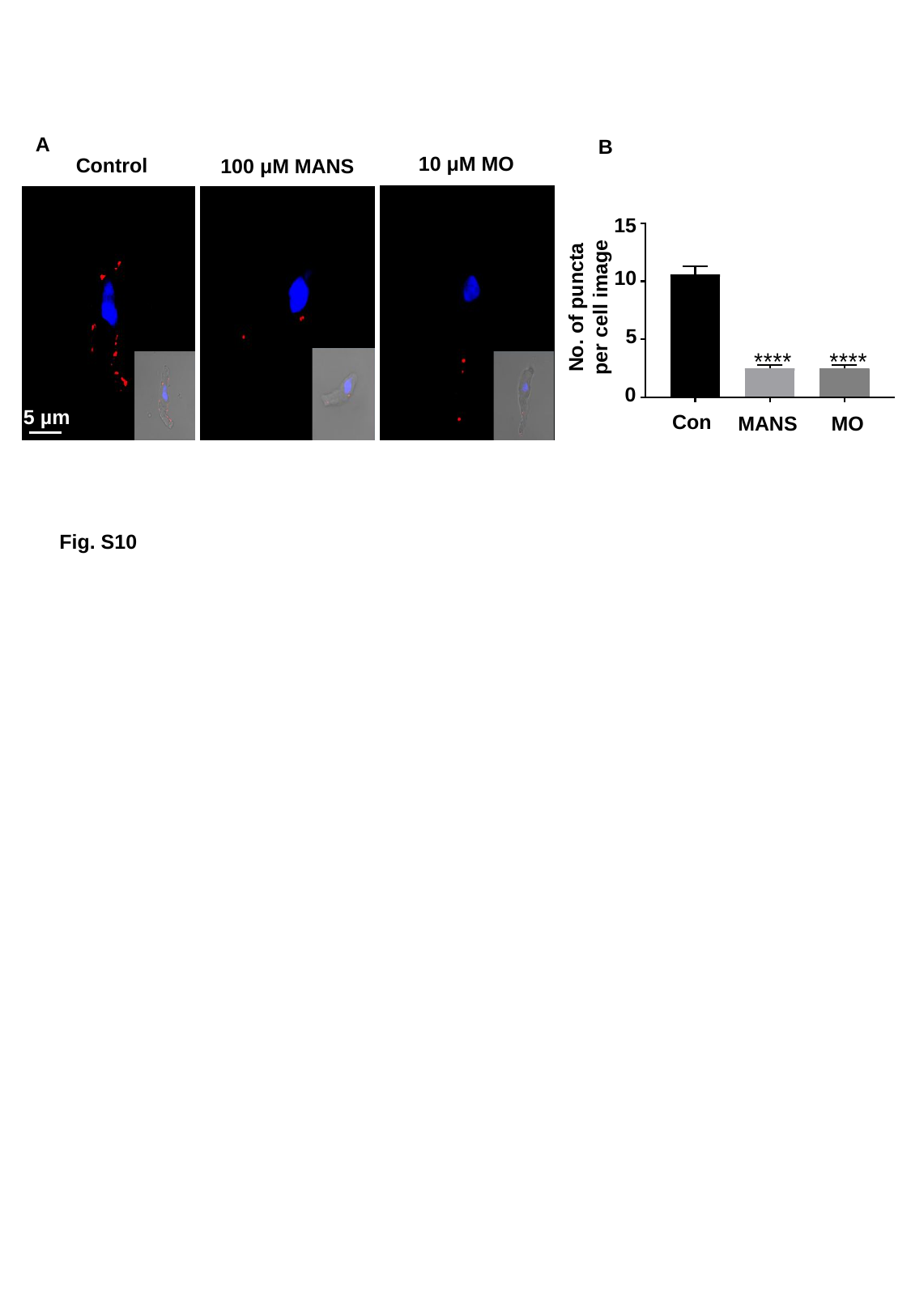

A
10 μM MO
Control
5 µm
100 μM MANS
B
15
10
5
0
No. of puncta
per cell image
Con
MANS
MO
Fig. S10

## Slide 11
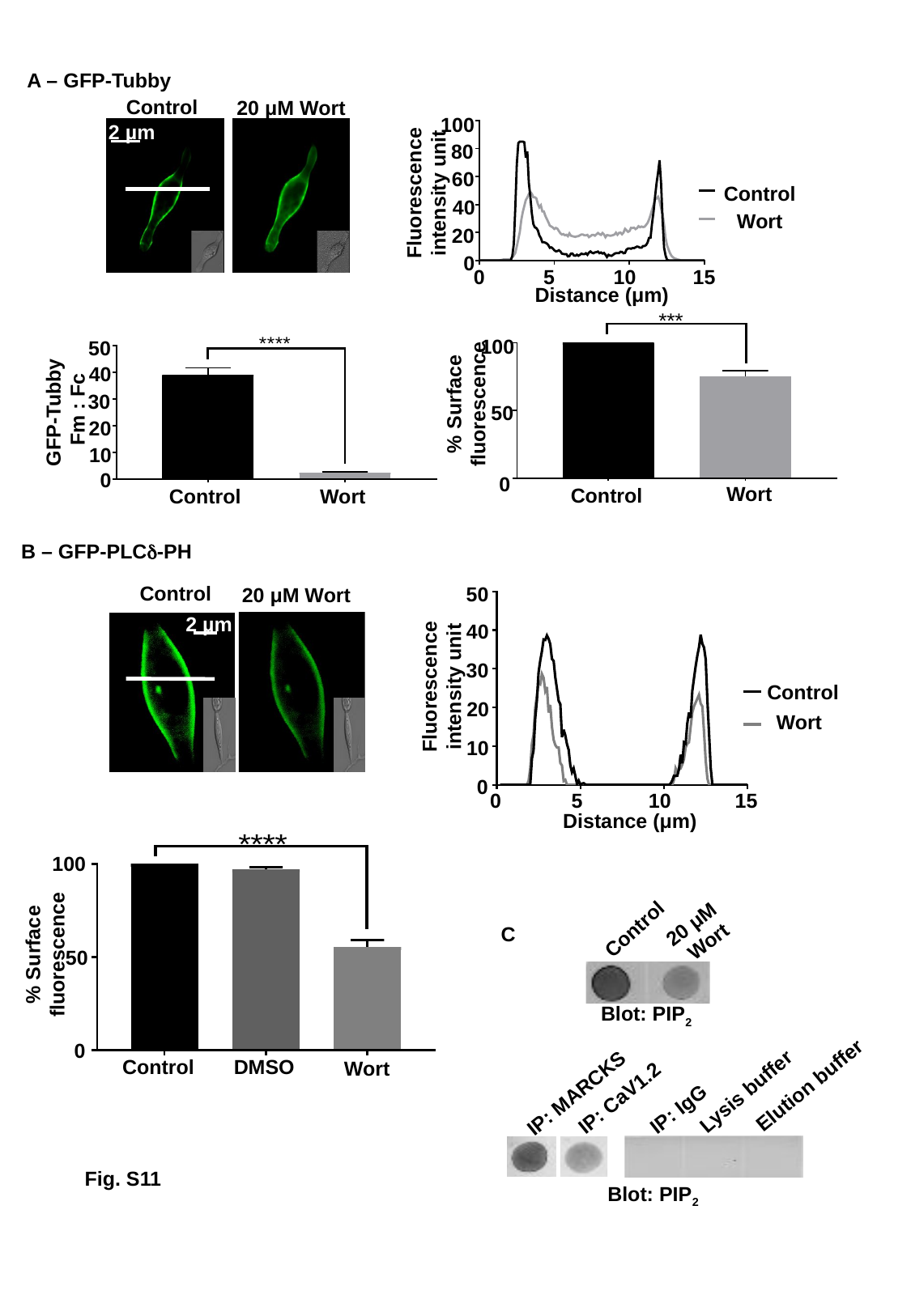

A – GFP-Tubby
Control
2 µm
20 μM Wort
100
80
60
Control
40
Wort
20
0
0
5
15
10
Distance (μm)
Fluorescence
intensity unit
100
% Surface
fluorescence
50
0
Wort
Control
50
40
30
GFP-Tubby
 Fm : Fc
20
10
0
Wort
Control
B – GFP-PLCδ-PH
Control
2 µm
20 μM Wort
50
40
Fluorescence
intensity unit
Control
20
0
15
5
0
10
Distance (μm)
30
Wort
10
100
% Surface fluorescence
50
0
Control
DMSO
Wort
20 μM
Wort
Control
Blot: PIP2
C
Elution buffer
Lysis buffer
IP: IgG
IP: MARCKS
IP: CaV1.2
Blot: PIP2
Fig. S11

## Slide 12
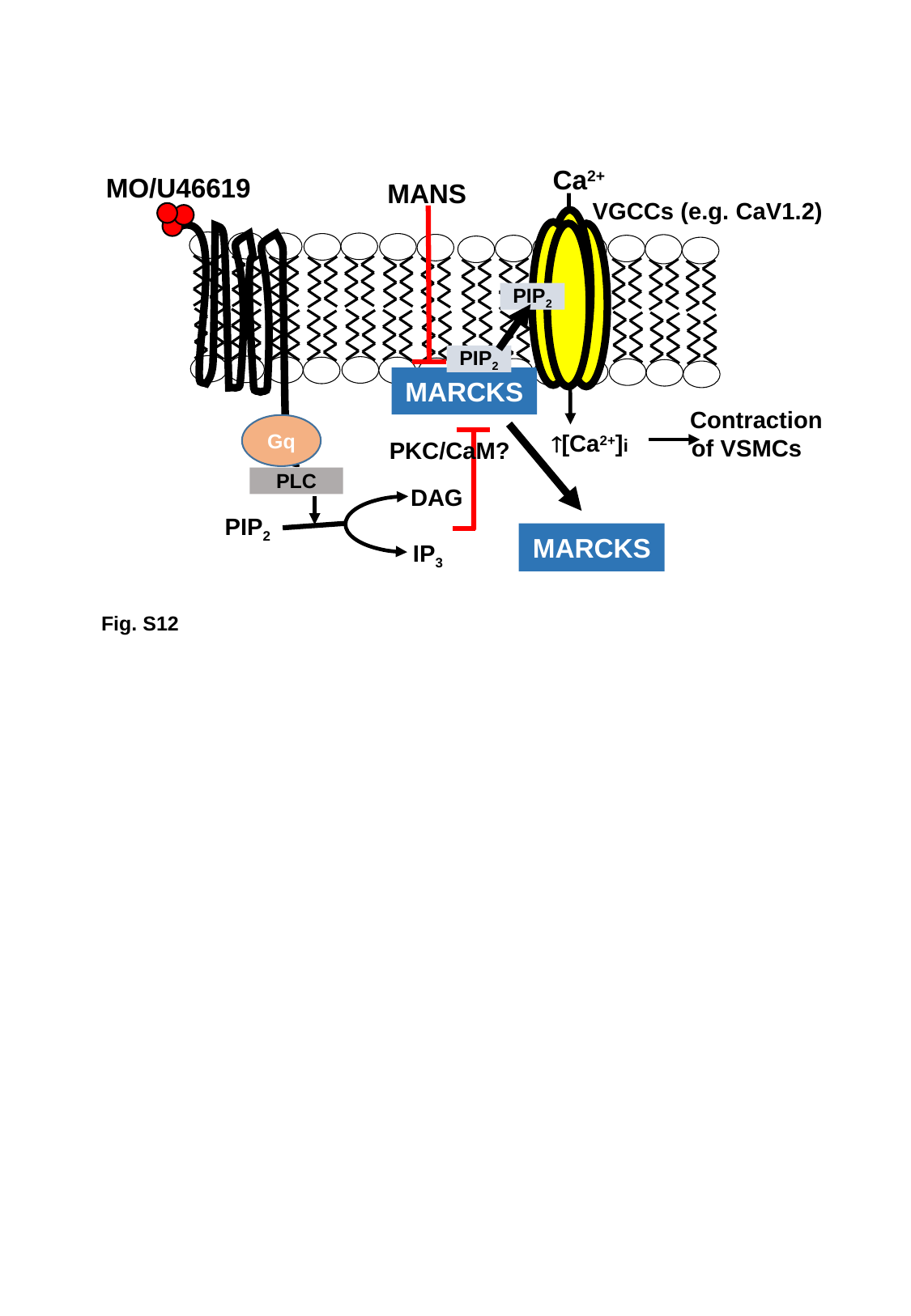

Ca2+
MO/U46619
MANS
VGCCs (e.g. CaV1.2)
PIP2
PIP2
MARCKS
Contraction
of VSMCs
[Ca2+]i
PKC/CaM?
PLC
DAG
PIP2
MARCKS
IP3
Gq
Fig. S12
