# Supplementary material for: MARCKS mediates vascular contractility through regulating interactions between voltage-gated Ca2+ channels and PIP2
Source: Vascul Pharmacol. 2020 Sep;132:106776. doi: 10.1016/j.vph.2020.106776 (PMC7549404; doi:10.1016/j.vph.2020.106776)
Supplement: Supplementary file 2 — Supplementary information [file mmc2.docx]

**Supplementary Information**

**Methods**

**Protein Extraction**

Cell lysates were extracted from mesenteric arteries arcades of mice or rats. Vessels were weighed, placed in 3 ml/g radio immunoprecipitation assay (RIPA) lysis buffer containing protease inhibitor cocktail (PI) (Santa Cruz, USA) and cut into smaller pieces. Vessels were homogenized using a pellet pestle for 5 min and sonicated for 20 min on ice. Vessels were then centrifuged at 15,000 x *g* for 20 min at 4°C. Supernatant was carefully transferred to a new eppendorf tube and quantified by performing a protein assay. RIPA lysis buffer contained: 150 mM NaCl, 1.0% (v/v) NP-40, 1.0% (v/v) Triton X-100, 0.5% (w/v) sodium deoxycholate, 0.1% (w/v) sodium dodecyl sulfate (SDS) and 50 mM Tris (pH 8.0).

**Western Blotting**

One-dimensional protein gel-electrophoresis was performed in 4-12% Bis-Tris gels in a Novex mini-gel system (Invitrogen, UK). Samples were mixed with Nu-Page LDS sample buffer (Life Technologies, UK), heated for 5 min at 95°C and run alongside a protein standard. 4-12% gels were run with NuPage MOPS-SDS running buffer (Life Technologies, UK) mixed with 500 µl NuPage antioxidant (Invitrogen, UK), at 200 mV for 50 min. Separated proteins were transferred onto a PVDF membrane (Life Technologies, UK) using iBlot transfer system at 20V for 7 min (Invitrogen, UK). Membranes were then immediately blocked in 5% (w/v) milk powder in PBS + 0.05% (v/v) Tween (PBST) for 1 h on a gyro-rocker at room temperature. Membranes were then incubated in anti-MARCKS (1:200; SC-6455, Santa Cruz, USA) antibody diluted in 5% (w/v) milk/PBST overnight at 4°C on a gyro-rocker. The next morning membranes were washed 3 times for 10 min at room temperature with PBST. Membranes were then incubated with a horseradish peroxidase-conjugated antibody diluted in milk/PBST for 1 h on a gyro-rocker at room temperature. Membranes were subsequently washed 4 times for 15 min in PBST on a gyro-rocker at room temperature before being treated with electrochemiluminescence (ECL) prime western blotting detection reagent (GE Healthcare, UK) for 1 min. Immunoreactive bands were visualized using photographic films (ThermoFisher Scientific, UK).

**VSMC Isolation**

Mesenteric arteries from mice or rats were enzymatically dispersed into single VSMCs by incubation in 0 mM Ca^2+^-DPSS with 0.5 mg/ml protease (Sigma, UK) for 5 min followed by incubation with 50 μM Ca^2+^-DPSS 1 mg/mL collagenase type IA (Sigma, UK) for 14 min at 37°C. Vessels were then washed in 50 μM Ca^2+^-DPSS for 10 min at 37°C and further incubated in 50 μM Ca^2+^-DPSS at room temperature for 10 min. Cells were then released into the solution by gently triturating the tissue using a fire polished wide-bore Pasteur pipette. The suspension of cells was then centrifuged at 1000 x *g* for 2 min to form a loose pellet that was subsequently re-suspended in 0.75 mM Ca^2+^-DPSS. Normal DPSS contained (mM): 126 NaCl, 6 KCl, 10 glucose, 11 HEPES, 1.2 MgCl_2_ and 1.5 CaCl_2_, with pH adjusted to 7.2 with 10 M NaOH. Low Ca^2+^-DPSS (0 mM, 50 μM and 0.75 mM) had the same composition as previously described, except that 1.5 mM CaCl_2_ was replaced by 0 mM, 50 μM and 0.75 mM CaCl_2_, respectively.

**Isometric Tension Recordings**

Segments of mouse superior mesenteric artery were mounted on a wire myograph and normalised. Vessel segments were equilibrated and assessed for vessel viability followed by endothelium integrity before one of five experimental protocols were performed.

Protocol 1- To investigate the effect of MANS on vascular contractility, increasing concentrations of MANS (1 nM-100 μM) (Gene-med synthesis, USA), a synthetic selective MARCKS inhibitor, was cumulatively added to vessel segments. These responses were compared to contractions produced by increasing concentrations of methoxamine (MO) (1 nM- 100 μM) (Sigma, UK).

Protocol 2-To examine whether contractions produced by MANS peptide were sensitive to VGCC blockers, increasing concentrations of various VGCC blockers or equivalent dilutions of their appropriate vehicle control were cumulatively added to vessel segments pre-contracted with 100 μM MANS.

Protocol 3-To determine whether contractions produced by MANS were sustainable, artery segments were contracted with 100 μM MANS for at least 30 min. These contractions were compared to contractions produced by 10 μM MO.

Protocol 4-To explore whether contractions produced by MANS were reproducible with multiple additions, artery segments were contracted with 100 μM MANS for at least 5 min. MANS was then washed out and artery segments were allowed to equilibrate for 10 min. Vessel segments were then subjected to a second contraction with 100 μM MANS. Artery segments were contracted three times in total and contractions produced by 100 μM MANS were compared to contractions by 10 μM MO.

**Transfection of PIP_2_ Biosensors**

Electroporation was performed using Nucleofector™ Technology (Lonza, USA). Rat mesenteric artery branches were enzymatically dispersed into single VSMCs and counted using a Countess^®^ automated cell counter (Invitrogen, UK). Next, 1x10^6^ cells were centrifuged at 100 x *g* for 10 min at room temperature. Cells were then re-suspended in 100 μl room temperature basic nucleofector kit primary smooth muscle cell solution (Lonza, USA) and combined with 2 μg plasmid DNA (GFP-PLCδ-PH or GFP-Tubby). Cell/DNA suspension was transferred into a supplied cuvette and the appropriate nucelofector programme (U-025) was selected. Following electroporation, 500 μl pre-warmed cell culture media was immediately added to the cell/DNA suspension. Samples were then seeded on a 96 well plate overnight at 37°C in 95% O_2_ and 5% CO_2_ in a humidified incubator. The following morning, cell culture media was replaced (with fresh cell culture media) and cells were incubated at 37°C in 95% O_2_ and 5% CO_2_ in a humidified incubator for a further 24 h before being imaged.

**Immunoprecipitation**

Immunoprecipitation was carried out using the Millipore Catch and Release kit (MERCK, UK), where spin columns were loaded with 500 μg of cell lysate protein and 4 μg of anti-MARCKS (1:100; SC-6455, Santa Cruz, USA) or anti-CaV1.2 (1:100; ACC-003, Alomone, Israel) primary antibody for 1 h at room temperature. Non-precipitated proteins were washed away with Millipore Catch and Release wash buffer and immunoprecipitated samples were then eluted with Millipore Catch and Release non-denaturing elution buffer. Immunoprecipitated samples were subsequently used for dot-blotting.

**Proximity Ligation Assays**

Interactions between MARCKS and CaV1.2 were studied with Duolink^®^ In Situ Red Starter Kit (Sigma, UK). Freshly dispersed mouse mesenteric VSMCs (unstimulated or pre-treated with various agonists or their vehicle controls) were placed on polylysine coated microscope slides and left to adhere for 1 h at room temperature before being fixed with 4% (w/v) PFA for 15 min. Cells were then rinsed with ice cold PBS twice for 10 min and permeabilized with PBS containing 0.10% (v/v) Triton X-100 for 10 min at room temperature. Cells were then washed with ice cold PBS, 3 times every 5 min, and incubated with Duolink blocking buffer for 1 h at 37°C. Cells were then incubated with anti-MARCKS (1:50; SC-6455, Santa Cruz, USA) and anti-CaV1.2 (1:50; ACC-003, Alomone, Israel) antibodies, diluted in Duolink antibody diluent solution, overnight at 4°C. The following morning cells were washed with ice cold PBS, twice for 5 min, and incubated with oligonucleotide conjugated secondary antibodies, diluted in Duolink antibody diluent solution (1:5), for 1 h at 37°C. Unbound secondary antibodies were removed by washing with Wash Buffer A twice for 2 min and cells were incubated in Ligation-Ligase Solution for 1 h at 37°C. Cells were then washed with Wash Buffer A twice for 2 min and incubated in Amplification-Polymerase Solution for 2 h at 37°C in the dark. Then cells were washed with Wash Buffer B twice for 10 min and 0.01x Wash Buffer B for 1 min and slides were left to dry at room temperature in the dark. Next, cells were treated with Duolink *In Situ* Mounting Medium with DAPI and cover slips were mounted to microscope slides. Cells were imaged using a Zeiss LSM 510 laser scanning confocal microscope (Carl Zeiss, Germany). Excitation was produced by 594 nm lasers and delivered to cells *via* a Zeiss Apochromat x63oil-immersion objective (numerical aperture, 1.4). Fluorescent puncta were captured using LSM 510 software (release 3.2; Carl Zeiss, Germany). The mean number of puncta per cell was calculated by counting the number of particles across a z-stack of the cell. Final images were produced using PowerPoint (Microsoft, USA). Control experiments were carried out by omitting both primary antibodies.

**IP_3_ ELISA**

IP_3_ levels were determined with a mouse IP_3_ ELISA kit (BlueGene Biotech, China) following the manufacturer’s instructions. Mouse mesenteric arteries were dissected, divided into three, and treated with vehicle, 100 μM MANS peptide, or 10 μM MO before being lysed as previously described. 100 μl standards or samples (in triplicate) were added to the appropriate wells. Next, 10 μl balance solution was added into 100 μl samples. Then 50 μl of conjugate was added to each well and the plate was incubated for 1 h at 37°C in the dark. The plate was then washed five times with 1 x wash buffer to remove unbound antibodies before being inverted and blot dried. Next, 50 μl substrate A and 50 μl substrate B were added to each well, sequentially, and the plate was incubated for 30 min at 37°C in the dark. Finally, 50 μl stop solution was added to each well and the absorbance reading at 450nm was determined using a microplate reader (SpectraMax 340PC384; Molecular Devices, USA).

**Figure legends**

**Fig. S1.** Characterisation of MANS-evoked contractions in mouse superior mesenteric artery segments.

A, Representative traces and mean data comparing sustainability of contractions induced by MO (left panel) and MANS (right panel). B, Representative traces and mean data comparing the reproducibility of contractions induced by MO (left panel) and MANS (right panel) with multiple additions. Data from *n*=3 animals, with N≥3 vessel segments per animal.

**Fig. S2.** Morpholino-induced MARCKS knock-down in mouse mesenteric artery tissue lysates.

A, Representative images showing increased fluorescence of artery segments transfected with fluorescein-tagged morpholino (right panel) compared with artery segments transfected with non-fluorescein-tagged morpholino (left panel). Representative western blots (B and C), and mean data comparing protein expression of MARCKS (D), α-tubulin (E) total protein (F), in tissue lysate from vessel segments pre-treated with scrambled or MARCKS-targeted morpholino. Data from N=6 experimental preparations with *n*=2 animals used for each preparation. Unpaired students *t*-test. **P*<0.05. ns indicates not significant.

**Fig. S3.** Morpholino-induced MARCKS knock-down on CaV1.2 levels in mouse mesenteric artery tissue lysates.

A and B, Representative western blot and mean data showing protein expression of CaV1.2 in tissue lysate from vessel segments pre-treated with scrambled or MARCKS-targeted morpholino. Data from N=6 experimental preparations with *n*=2 animals used for each preparation. Unpaired students *t*-test. **P*<0.05. ns indicates not significant.

**Fig. S4.** Expression and localisation of MARCKS and CaV1.2 in single mouse mesenteric VSMCs following MARCKS knock-down.

A, Representative images of scrambled (top panel) or MARCKS-targeted (bottom panel) morpholino pre-treated VSMCs co-immunolabelled with anti-MARCKS (green) and anti-CaV1.2 (red) antibodies. B and C, Representative and mean data of line scans showing anti-MARCKS (left panel) and anti-CaV1.2 (right panel) signals across the cell width and graphs showing fluorescent intensities at the cell surface in scrambled and MARCKS-targeted morpholino pre-treated VSMCs. Data from *n=*4 animals with N≥6 cells per animal. Unpaired students *t*-test. *****P*<0.0001. ns indicates not significant.

**Fig. S5.** Effect of MARCKS knock-down on resting tension and evoked contractions.

Tables showing mean data comparing resting tension (A), and MANS- (B), methoxamine (MO)- (C) and U46619-evoked contractions (D) in scrambled and MARCKS-targeted morpholino treated vessels. Data from *n*=6 animals, with N=4 segments per animal. Two-way ANOVA. *****P*<0.0001.

**Fig. S6.** Effect of MARCKS knock-down on KCl-and ionomyocin-induced contractions in mouse mesenteric arteries.

A and B, Representative traces and mean data comparing contractions induced by KCl (top panel) and ionomyocin (bottom panel) in scrambled and MARCKS-targeted morpholino oligonucleotides treated vessels. Data from *n=*4 animals, with N≥4 segments per animal.

**Fig. S7.** Table comparing the mean data of L-type and T-type VGCC blockers on MANS pre-constricted tone in mouse mesenteric artery segments.

**Fig 8.** Effect of MANS and methoxamine (MO) on GFP-tubby signals in single rat mesenteric artery vascular smooth muscle cells.

A, Representative image from a single cell showing that in control conditions, the location of GFP-tubby-mediated signals was predominantly expressed at the plasma membrane. In the same cell, application of MANS had no significant effect on GFP-tubby-mediated signals while subsequent treatment with MO induced translocation of signals to the cytosol. B, Line scans showing GFP-tubby signals across the cell width in control conditions, following treatment with MANS and subsequent application of MO. C, Mean data showing GFP-tubby Fm:Fc ratios in control conditions, treatment with MANS, followed by application of MO. Data from *n*=6 animals, with N≥4 cells per animal. Paired students *t*-test. *****P*<0.001. ns indicates not significant.

**Fig. S9.** Comparison of IP_3_ levels in mouse mesenteric artery tissue lysates from vessel segments pre-treated with MANS or methoxamine (MO).

Mean data showing that pre-treating vessel segments with MANS had no significant (ns) effect on relative IP_3_ levels in tissue lysate. Whereas, pre-treating segments with MO increased relative IP_3_ levels in tissue lysate. Data from N=3 experimental preparations with *n*=3 animals used per preparation. One-way ANOVA. *****P*<0.0001. ns indicates not significant.

**Fig. S10.** Interaction between MARCKS and CaV1.2 in single mouse mesenteric artery VSMCs.

Representative proximity ligation assay (PLA) images of single VSMCs (A) and mean data (B) which show that in control cells MARCKS and CaV1.2 interact at the plasma membrane. Pre-treatment with MANS or methoxamine (MO) reduced interactions between MARCKS and CaV1.2. Data from *n*=6 animals, with N=≥6 cells per animal. One-way ANOVA. *****P*<0.0001.

**Fig. S11.** Effect of wortmannin on PIP_2_ levels in VSMCs.

A, Representative images of a single GFP-tubby transfected rat mesenteric artery VSMC, line scan, and mean data showing that wortmannin (Wort) reduced GFP-tubby signals at the plasma membrane and caused translocation of GFP-tubby signals to the cytosol. Data from *n*=4 animals, with N≥4 cells per animals. B, Representative image of a single GFP-PLCδ-PH transfected rat mesenteric artery VSMC, line scan, and mean data showing that wortmannin reduced GFP-PLCδ-PH- signals at the plasma membrane. Data from *n*=4 animals, with N≥4 cells per animal. C, Representative dot-blot with an anti-PIP_2_ antibody which shows that wortmannin reduced PIP_2_ fluorescence intensity (top panel) and that immunoprecipitation with a non-specific IgG or blotting with lysis or elution buffer (bottom panel) produced no fluorescence. Data from N=3 preparations with *n*=3 animals used per preparation. Paired students *t*-test. ****P*<0.001; *****P*<0.0001.

**Figure S12.** Proposed signalling pathway coupling Gq-receptor stimulation and MARCKS to regulation of vascular contractility.

The present work indicates that MARCKS regulates vascular contractility by modulating voltage-gated Ca**^2+^** channel (VGCC) activity. We propose that in resting, unstimulated vascular smooth muscle cells (VSMCs), MARCKS associates with VGCCs to inhibit contractility by sequestering local PIP_2_ levels, reducing PIP_2_-mediated facilitation of VGCC activity. Disinhibition of MARCKS (represented by the red lines) by MANS or stimulation of Gq-coupled receptors by vasoconstrictors (e.g. MO and U46619) leads to dissociation of MARCKS-VGCC interaction and translocation of MARCKS to the cytosol which causes release of this sequestered PIP_2_ at the plasma membrane that binds to, and facilitates VGCC activity, to promote vasoconstriction. The pathway linking Gq-coupled receptors to MARCKS is unknown but is likely to involve protein kinase C (PKC) and/or calmodulin (CaM).
